# Divergence thresholds and divergent biodiversity estimates: can metabarcoding reliably describe zooplankton communities?

**DOI:** 10.1002/ece3.1485

**Published:** 2015-05-13

**Authors:** Emily A Brown, Frédéric J J Chain, Teresa J Crease, Hugh J MacIsaac, Melania E Cristescu

**Affiliations:** 1 Department of Biology, McGill University1205 Docteur Penfield, Montreal, Quebec, Canada, H3A 1B1; 2 Great Lakes Institute for Environmental Research, University of WindsorWindsor, Ontario, Canada, N9B 3P4; 3 Department of Integrative Biology, University of Guelph50 Stone Road East, Guelph, Ontario, Canada, N1G 2W1

**Keywords:** Biodiversity, high-throughput sequencing, metabarcoding, mock community, nSSU, zooplankton

## Abstract

DNA metabarcoding is a promising method for describing communities and estimating biodiversity. This approach uses high-throughput sequencing of targeted markers to identify species in a complex sample. By convention, sequences are clustered at a predefined sequence divergence threshold (often 3%) into operational taxonomic units (OTUs) that serve as a proxy for species. However, variable levels of interspecific marker variation across taxonomic groups make clustering sequences from a phylogenetically diverse dataset into OTUs at a uniform threshold problematic. In this study, we use mock zooplankton communities to evaluate the accuracy of species richness estimates when following conventional protocols to cluster hypervariable sequences of the V4 region of the small subunit ribosomal RNA gene (18S) into OTUs. By including individually tagged single specimens and “populations” of various species in our communities, we examine the impact of intra- and interspecific diversity on OTU clustering. Communities consisting of single individuals per species generated a correspondence of 59–84% between OTU number and species richness at a 3% divergence threshold. However, when multiple individuals per species were included, the correspondence between OTU number and species richness dropped to 31–63%. Our results suggest that intraspecific variation in this marker can often exceed 3%, such that a single species does not always correspond to one OTU. We advocate the need to apply group-specific divergence thresholds when analyzing complex and taxonomically diverse communities, but also encourage the development of additional filtering steps that allow identification of artifactual rRNA gene sequences or pseudogenes that may generate spurious OTUs.

## Introduction

Metabarcoding has become a well-established tool for the rapid assessment of biodiversity. The combination of DNA-based identification (barcoding) with high-throughput sequencing (HTS) renders this method particularly useful when examining cryptic biodiversity in complex ecosystems. The massively parallel nature of HTS technologies provides extensive sequencing depth (Buée et al. 2009; Tedersoo et al. 2010; Blaalid et al. 2012; Davey et al. 2012), which increases the chance of obtaining data for species that occur at low abundances (Jerde et al. 2011; Diaz et al. 2012; Zhan et al. 2013). Metabarcoding has been applied to identify various groups of organisms including soil microbes (Shade et al. 2012), freshwater protists (Bråte et al., 2010), and aquatic metazoans (Fonseca et al. 2010), among others. Many such studies have revealed estimates of biodiversity orders of magnitude higher than those previously generated with traditional methods (Sogin et al. 2006; Stoeck et al. 2009; Bachy et al. 2013; Lindeque et al. 2013), giving rise to the concept of the “rare biosphere” (Pedrós-Alió 2007). While these findings might be explained by the demonstrated high sensitivity of HTS-based techniques (Jerde et al. 2011; Zhan et al. 2013), concern has been raised over the accuracy of biodiversity estimates generated through metabarcoding (Reeder and Knight 2009; Huse et al. 2010; Quince et al. 2011).

A number of technical considerations are associated with metabarcoding, some of which are better understood than others (Cristescu 2014). For example, it is well recognized that the unprecedented amounts of sequencing data generated by HTS are not error free (Huse et al. 2007). Pyrosequencing is frequently used in metabarcoding studies because it generates relatively long reads, which are often necessary to distinguish species, but it does have a high error rate in homopolymer regions (Margulies et al. 2005). Metabarcoding also involves PCR amplification of a “barcode” region, which can introduce noise into the data as polymerases do not replicate DNA perfectly, and chimeric sequences can form (Meyerhans et al. 1990; Gaspar and Thomas 2013). Numerous studies have examined the various programs and algorithms developed for filtering HTS datasets, with the aim to filter out errors and artifacts that were introduced during sequencing and PCR (Schloss et al. 2011; Sun et al. 2012; Gaspar and Kelley Thomas 2013). A simple approach, often applied in conjunction with additional quality filters, excludes singletons (sequences that occur only once) from datasets, as erroneous and artifactual sequences are likely to be generated during a single random event. Quality-filtered reads are often grouped at a user-defined sequence divergence threshold into clusters known as operational taxonomic units (OTUs) in order to characterize the taxonomic composition of a PCR-amplified community (Bonder et al. 2012). However, even after extensive quality filtering, spurious OTUs may still be produced (Quince et al. 2009; Kunin et al. 2010; Behnke et al. 2011).

Many of the metabarcoding studies carried out to date have focused on the amplification of hypervariable regions of the small subunit (SSU) rRNA genes, with sequences more than 3% divergent often recognized as belonging to different OTUs (Sogin et al. 2006; Huber et al. 2007; Stoeck et al. 2009). The V4 domain is the largest variable region of the eukaryotic SSU (18S) rRNA gene (Hadziavdic et al. 2014) and has been used to reveal the composition of complex eukaryote communities (Lindeque et al. 2013; He et al. 2014). Eukaryotic rRNA genes are organized in tandemly repeated arrays within a genome (Bik et al. 2012), and the number of gene copies can vary by several orders of magnitude across taxa (Prokopowich et al. 2003; Zhu et al. 2005). The number of rRNA gene copies can also vary within species (Averbeck and Eickbush 2005; Eagle and Crease 2012), and intragenomic variation can be extensive (James et al. 2009; Ambrose and Crease 2011). The V4 domain of the 18S gene not only has high nucleotide substitution rates, but also high indel rates (Wuyts et al. 2000). Depending on the clustering divergence threshold applied, sequence variants originating from a single genome/species might form multiple OTU clusters and thus be interpreted as representing distinct species, thereby inflating biodiversity estimates. This becomes particularly problematic when using HTS methods that may be capable of detecting even low-frequency 18S copies (Lindner et al. 2013). Intraspecific variation in 18S may complicate the correlation between OTU number and species richness, and variation in levels of intraspecific and interspecific variation across species will make using a uniform divergence threshold problematic, especially when examining a phylogenetically diverse community.

Without empirical data, there is no objective way to select the algorithm or input parameters that best recover actual species boundaries. Previous studies have shown that the divergence threshold applied when clustering sequences into OTUs has a large impact on the number of OTUs generated (Fonseca et al. 2010; Behnke et al. 2011; Egge et al. 2013). However, if the number of species present within a community is not known a priori, it is difficult to know which threshold most accurately estimates species richness. Artificially assembled or mock communities provide a solution to this problem, as the identity and number of species contained within the community is known. For example, Behnke et al. (2011) used a mock protistan community to show that diversity can be overestimated up to threefold even when clustering sequences at the commonly accepted 3% divergence threshold. Behnke et al. (2011) also found that the divergence threshold necessary to best reflect true diversity varied across taxon groups, with clustering at 4% sequence divergence accurately reflecting the number of ciliate species, but clustering at 9% still overestimating rhizarian diversity. More recently, Decelle et al. (2014) investigated intracellular diversity within 18S, but as did Behnke et al. (2011), examined only protists. Given the widespread use of highly variable markers such as 18S, it is imperative to understand the limitations inherent in HTS of rRNA gene amplicon libraries before undertaking large-scale studies to answer ecological or health-related questions about species diversity (Diaz et al. 2012). Many urgent conservation projects rely on accurate biodiversity estimates and would be greatly advanced by extensive metabarcoding studies that assess genetic variation across a broad range of metazoan groups and markers. Such studies would allow the estimation of interspecific variation and the application of group-specific thresholds when OTU clustering. Moreover, thorough examination of genetic variation within markers would allow evaluation of marker efficiency – if species are to be readily distinguished, intraspecific variation must be consistently lower than interspecific variation (i.e., a barcoding gap (Hebert et al. 2003) must be present). However, the extent of intraspecific and intragenomic variation in metabarcoding markers is often unknown.

In this study, we examine the correspondence between OTU number and species richness in mock communities of zooplankton while following conventional OTU clustering procedures. Specifically, we explore levels of intra- and interspecific divergence within these communities using pyrosequencing of 18S V4 amplicons and evaluate the clustering threshold necessary for the accurate estimation of species richness across diverse taxonomic groups. We apply a new approach that allows the sequences generated by the species or taxonomic groups present in our communities to be easily identified. This approach enables the separation and independent examination of sequences generated by single individuals or multiple individuals (“populations”).

We constructed four complex zooplankton communities consisting of species present either as single individuals or as “populations” at different densities. Each individual and population was individually tagged by incorporating different short sequence motifs in the primers. We included populations of different sizes to examine whether the same divergence threshold can be applied to samples with elevated levels of intraspecific variation. As far as we know, this is the first time that complex mock communities with multiple layers of genetic variation have been independently tagged and used to validate OTU estimates.

Although the use of tagged primers facilitates the examination of intra- and interspecific variation, individuals in natural communities are mass-DNA-extracted and thus cannot be tagged individually. Therefore, we also examined “untagged” communities, that is, DNA templates containing individuals of multiple species that were not amplified with tagged primers. For each tagged individual, tagged population, and untagged community, we tested whether the OTUs generated correspond to the expected species. As levels of intraspecific variation may vary across species, we also test whether a uniform divergence threshold is appropriate when clustering sequences generated from a phylogenetically diverse community into OTUs.

## Materials and Methods

### Assembly of the mock communities

Four mock zooplankton communities were constructed in order to evaluate the intraspecific and interspecific divergence levels within the V4 region of 18S across various taxonomic groups. The mock communities included species drawn from broad taxonomic groups including representatives of Mollusca, Tunicata, Rotifera, and six crustacean taxa (Amphipoda, Anostraca, Cladocera, Cirripedia, Copepoda, and Decapoda). These specimens were identified to the species or genus level by taxonomists, with a few exceptions that were identified to the family level. Two communities, hereafter referred to as “Tagged Individuals Community” and “Tagged Populations Community,” consisted of either single individuals of 20 species (Table S1) or populations of 13 species (Table S2), respectively, that were separately PCR-amplified with tagged primers. An additional two communities consisted of either single individuals of 61 species (Table S3) or populations of 14 species (Table S4) that were mass-DNA-extracted and PCR-amplified together without the use of tagged primers (i.e., Untagged Individuals Community and Untagged Populations Community). The inclusion of single individuals in the Individuals Communities allowed examination of intragenomic variation, with the expectation that each individual returns a single OTU. The Populations Communities allowed examination of intraspecific variation, as each species was represented by multiple individuals (or “populations”). Including multiple species in both the Individuals and Populations Communities also allowed examination of interspecific variation.

Samples of individuals and populations were prepared in microcentrifuge tubes and stored at −20**°**C. Many species were preserved in 95% ethanol, in which case they were sequentially washed in sterile distilled water prior to DNA extraction to remove ethanol and contaminants, such as algae and other zooplankton. Live animals were similarly washed to remove contaminants. Whole individuals of small animals such as copepods and *Daphnia* were used. We were careful to ensure that brooding animals were not used, and where possible, males were selected. Larger animals such as *Leptodora kindtii* and adult *Corbicula fluminea* were dissected to yield a small piece of tissue with roughly equivalent volume as that of an adult daphniid. Once the individuals/populations/communities were assembled in tubes, any fluid remaining from the washing process was removed by centrifugation at 6797 g for 3 min. The supernatant was subsequently examined under the microscope to ensure that no animals or tissue were lost during this process.

### Tagged individuals community

Single individuals of 20 zooplankton species (Table S1) were independently DNA-extracted and amplified with unique tagged primers (see below for information on DNA extraction, PCR, and cleaning protocols). Cleaned PCR products of these individuals were quantified and pooled such that each individual was present at equimolar concentrations.

### Untagged individuals community

A single individual (or part of an individual) from 61 zooplankton species of eight taxonomic groups (Table S3) was included in this community. As Qiagen recommends against overloading their DNeasy spin columns, the community was assembled in four separate microcentrifuge tubes, each containing between 14 and 16 individuals. These four subcommunities were DNA-extracted, PCR-amplified with the same primer pair, and pooled to form the Untagged Individuals Community, with the aim of equal representation for each species.

### Tagged populations community

In total, 24 “populations” – multiple individuals of a single species collected from a single location – were separately DNA-extracted and amplified using tagged primers (Table S2). These populations came from six of the taxonomic groups investigated in this study. For some groups, “low” (∽five individuals), “medium” (∽10 individuals), and “high” (∽30 individuals) populations were included. PCR products were pooled together, with each individual represented by an equal amount of DNA. For example, a population of 10 individuals contained twice the amount of DNA compared to a population of five individuals.

### Untagged populations community

A total of 76 individuals from 14 zooplankton species (Table S4) were combined, with these species present at different densities (between 1 and 23 individuals). As with the Untagged Individuals Community, this community was assembled in four separate microcentrifuge tubes, each containing between 17 and 23 individuals. These subcommunities were DNA-extracted and PCR-amplified with the same primer pair, before being pooled to form the Untagged Populations Community.

## DNA extraction, PCR amplification, and pyrosequencing

Total genomic DNA was isolated using DNeasy Blood and Tissue Kits (Qiagen, Venlo, Limburg, Netherlands) following the manufacturer's protocol. The quality and quantity of each DNA extraction were assessed using Quant-iT PicoGreen dsDNA Assay kit (Invitrogen, Carlsbad, CA, USA). Approximately 400–600 bp of the V4 region of the 18S gene was amplified using a primer pair developed by Zhan et al. (2013) (Uni18S: AGGGCAAKYCTGGTGCCAGC; Uni18SR: GRCGGTATCTRATCGYCTT). Each PCR mixture (25 *μ*L) consisted of approximately 100 ng of genomic DNA, 1× PCR buffer, 2 mmol/L of Mg^2+^, 0.2 mmol/L of dNTPs, 0.4 *μ*mol/L of each primer, and 2 U of *Taq* DNA polymerase Genscript, Piscataway, NJ, USA). PCR cycling parameters consisted of an initial denaturation step at 95°C for 5 min, followed by 25 amplification cycles of 95°C for 30 sec, 50°C for 30 sec, 72°C for 90 sec, and a final elongation step at 72°C for 10 min. In order to reduce the effect of PCR biases that may have occurred in any given reaction, each of the four untagged subcommunities was PCR-amplified eight times, each individual of the Tagged Individuals Community was amplified twice, and each population of the Tagged Populations Community was amplified three times. The number of independent amplifications performed was scaled roughly to the number of individuals included in each reaction. Equimolar aliquots of each replicate were then pooled for sequencing.

**Figure 1:**
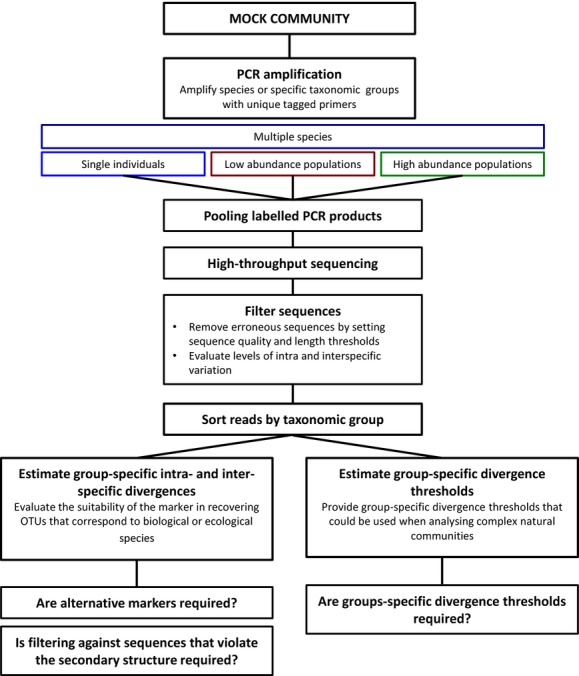
The use of complex mock communities that involve tagged primers to allow the separation and independent analysis of the sequences generated by different species or taxonomic groups. This method facilitates the identification of intra- and interspecific divergence levels. It also allows researchers to calibrate the thresholds of sequence divergence for all targeted taxonomic groups.

To ensure sample recognition in downstream analyses in the Tagged Communities, each individual and population was amplified with tagged primers – the forward primer was tagged specifically for each sample using unique 10-bp tags (MID sequences) approved by Roche (Technical bulletin 005-2009, Roche Diagnostics Corp., Basel, Switzerland) (Fig.1). For all primers, including those untagged, Roche 454 adaptors were attached in order to make them compatible with pyrosequencing procedures. All PCR products were cleaned using the solid-phase reversible immobilization (SPRI) paramagnetic bead-based method (ChargeSwitch, Invitrogen). Cleaned PCR products were quantified using gel electrophoresis and PicoGreen and pooled together as described above. Finally, two samples were prepared for pyrosequencing at the ½ PicoTiter plate scale – the Tagged and Untagged Individuals Communities were pooled to form one sample, and the Tagged and Untagged Population Communities were pooled to form the other. We aimed to have each individual within the two pools at equimolar concentration. Pyrosequencing was performed using 454 FLX Adaptor A on a GS-FLX Titanium platform (454 Life Sciences, Branford, CT, USA) by Genome Quebec.

## Data analysis

Raw sequence reads were analyzed using the UPARSE algorithm, implemented in USEARCH version 7.0.1090 (Edgar 2013). UPARSE was previously tested on a subset of our dataset (Flynn et al. 2015; THIS ISSUE) and was found to outperform alternative clustering algorithms (mothur and UCLUST) in terms of the accuracy of the species richness estimates generated. Reads with sequencing errors in the forward primer and tag were removed from the dataset using the python scripts provided with UPARSE. These scripts also trim the sequences at the primer sites such that the forward primer and tag sequences are removed. Reads were then trimmed to 400 bp, as not all sequences reached the reverse primer and sequence quality generally decreased beyond 400 bp (see Fig. S1). Sequences were also quality-filtered using a maximum expected error threshold of 0.5. The resulting reads were dereplicated (collapsed to unique sequences) and then clustered into operational taxonomic units (OTUs) using the UPARSE-OTU algorithm. The way that gaps in sequences are treated during OTU clustering is important when examining sequences that are prone to indels (Flynn et al. 2015, THIS ISSUE). For example, the UPARSE-OTU algorithm expects globally alignable sequences and by default treats each gap as an independent mutational event when calculating divergence between sequences. Therefore, terminal gaps created after sequence alignment during the OTU clustering affect divergence estimates. However, given the size of the datasets analyzed and the substantial length variation in V4, it is not feasible to conduct sequence alignments prior to sequence trimming and OTU clustering, as the accuracy of the alignment would be questionable.

Reads that occur within pyrosequencing datasets only once (“singletons”) are often considered to be artifacts, but it is also possible that they are low-abundance biological variants that were only sampled once from the pool of DNA. Thus, we present results from analyses in which singletons were either included or excluded from the data. Reads were clustered with sequence divergence thresholds from 1% to 10%. Although chimera detection is incorporated into the UPARSE clustering algorithm, we also used the algorithm UCHIME (Edgar et al. 2011), implemented in USEARCH version 7.0.1090, to remove remaining putative chimeras.

For taxonomic annotation, we performed a local BLAST (Altschul et al. 1990) search of each OTU (using the representative sequence as determined by UPARSE) versus local reference databases. The local reference databases were constructed for each community by downloading 18S sequences for each species in the community from the NCBI nucleotide database and the SILVA database (Quast et al. 2013). If the sequence for a particular species was not present within either of these databases, but that species could clearly be distinguished from others within the community (i.e., a species distantly related to other community members, as determined phylogenetically), we obtained the sequence of the closest available congeneric species. In cases where closely related species in the community needed to be distinguished, we used Sanger sequencing to generate an 18S V4 reference sequence that was added to our local BLAST database (Table S5). Some animals included in the community were only identified to the family level (e.g., Decapod larvae), in which case we either used Sanger sequencing to generate a reference sequence or included the sequence of a confamilial species from the NCBI database. The best BLAST hit against our local database was used to classify each sequence, and a positive identification was defined as a hit with at least 90% identity and an alignment length of at least 330 nucleotides with a database sequence. Although most hits returned >97% match (often a perfect match) with the reference sequence, these relatively relaxed thresholds were used to accommodate congeneric (or family level) reference sequences. In order to confirm which species were successfully amplified and sequenced within our communities, we also BLASTed all unfiltered reads against our local reference database. These unfiltered reads were raw reads that were only trimmed to remove the primer and tag regions, a process which also removed reads with sequence mismatches in these regions. Dendrograms of OTUs were generated using FastTree v 2.1.7 (Price et al. 2010).

## Results

### Individuals community

A total of 610,914 raw sequences were generated for the pooled Tagged and Untagged Individuals Communities. Filtered sequences were subsequently separated into two datasets corresponding to the Tagged and Untagged Communities (totaling 115,902 and 430,845 sequences after removal of barcode and primer errors, respectively). A total of 8,736 and 20,487 unique sequences remained following dereplication of each respective community (Table1).

**Table 1:** The number of raw reads generated by the two 454 sequencing runs (the Individuals and Populations Communities). Each run included both the Tagged and Untagged Communities.

	Individuals community		Populations community	
	610,914		625,239	
Raw reads	Tagged	Untagged	Tagged	Untagged
Barcode/primer error-filtered reads	115,902	430,845	404,052	199,871
Quality-filtered reads	58,334	229,435	296,944	142,969
Unique reads including singletons	8736	20,487	32,745	20,077
Singletons	6338	13,575	22,181	14,102
Unique reads excluding singletons	2398	6730	10,564	5975

#### Tagged individuals community

As each tag represented a different species and the data generated by each species were expected to form distinct OTUs, the filtered data encompassing 58,334 sequences for all 20 tagged individuals were clustered together (Table2). Sequences derived from one individual (*Dreissena polymorpha*) were removed from further analysis because its OTUs did not return a BLAST hit against this species, most likely as a result of contamination. With the 3% divergence threshold and singletons removed, clustering generated a single OTU for 16 of 19 (84%) tagged individuals, indicating a reasonable approximation of the number of known species in the dataset. *Corbicula fluminea* and *Palaemonetes* spp. generated two OTUs at the 3% threshold. Including singletons within the dataset resulted in additional OTUs for several species (*Artemia salina*, *Cercopagis pengoi*, *Ciona intestinalis*, *Corbicula fluminea*, *Palaemonetes* spp.). Conversely, the rotifer *Brachionus calyciflorus* was only identified when singletons were included in the dataset. This OTU was comprised of four singletons, demonstrating that singletons may allow detection of species at low abundance within communities.

**Table 2:** OTUs generated after clustering the data for the Tagged Individuals Community using a 3% divergence threshold. Results are reported when singletons are excluded and included. The number of filtered reads that were clustered to form each OTU is reported.

Tagged individual	Singletons excluded			Singletons included		
	No. OTUs	Species matching OTU(s)	No. reads in OTU	No. OTUs	Species matching OTU(s)	No. reads in OTU
*Artemia salina*	1	*Artemia salina*	795	2	*Artemia salina*	962
					*Artemia salina*	2
*Balanus crenatus*	1	*Balanus crenatus*	6231	1	*Balanus crenatus*	6943
*Brachionus calyciflorus*				1	*Brachionus calyciflorus*	4
*Cancer spp*.	1	*Cancer spp*.	418	1	*Cancer spp*.	544
*Cercopagis pengoi*	1	*Cercopagis pengoi*	259	2	*Cercopagis pengoi*	327
					*Cercopagis pengoi*	1
*Ciona intestinalis*	1	*Ciona intestinalis*	726	2	*Ciona intestinalis*	1057
					*Ciona intestinalis*	1
*Corbicula fluminea*	2	*Corbicula fluminea*	23,420	4	*Corbicula fluminea*	25,497
		*Corbicula fluminea*	3874		*Corbicula fluminea*	3883
					*Corbicula fluminea*	24
					*Corbicula fluminea*	1
*Daphnia mendotae*	1	*Daphnia mendotae*	62	1	*Daphnia mendotae*	84
*Diacyclops thomasi*	1	*Cyclops spp*.	158	1	*Cyclops spp*.	213
*Echinogammarus ischnus*	1	*Dikerogammarus villosus*	2947	1	*Dikerogammarus villosus*	3324
*Epischura lacustris*	1	*Epischura lacustris*	4209	1	*Epischura lacustris*	4969
*Leptodiaptomus ashlandi*	1	*Leptodiaptomus ashlandi*	1764	1	*Leptodiaptomus ashlandi*	2107
*Mesocyclops edax*	1	*Mesocyclops edax*	6	1	*Mesocyclops edax*	9
*Microsetella norvegica*	1	*Microsetella norvegica*	215	1	*Microsetella norvegica*	259
*Oikopleura labradoriensis*	1	*Oikopleura labradoriensis*	240	1	*Oikopleura labradoriensis*	375
*Palaemonetes* spp.	2	*Palaemonetes pugio*	3050	3	*Palaemonetes pugio*	3437
		*Palaemonetes pugio*	6		*Palaemonetes pugio*	9
					*Palaemonetes pugio*	1
*Pleuroxus denticulatus*	1	*Pleuroxus truncatus*	76	1	*Pleuroxus truncatus*	102
*Themisto libellula*	1	*Themisto libellula*	2928	1	*Themisto libellula*	3383
*Senecella calanoides*	1	*Senecella calanoides*	26	1	*Senecella calanoides*	39

We examined the threshold necessary to generate a single OTU in individuals that were represented by more than one OTU. When singletons were excluded, clustering at 4% divergence generated a single OTU for *Corbicula fluminea*, whereas 8% was necessary when singletons were included. Further examination of the representative sequences of the OTUs generated at 3% for *Corbicula* (when singletons are excluded) revealed that these sequences differed by 7-base pair (bp) substitutions and 4 small (1–6 bp) indels. A 10% divergence threshold was required to generate a single OTU for *Palaemonetes* spp. when singletons were included or excluded. Examination of the *Palaemonetes* OTU sequences showed that the high sequence divergence was caused by a single indel of 36 bp, each position of which was treated as a difference by the UPARSE-OTU algorithm during divergence estimation. When singletons were included, *Artemia salina, Cercopagis pengoi,* and *Ciona intestinalis* required divergence thresholds of 4%, 10%, and 8%, respectively, to generate a single OTU. Clustering the entire tagged dataset both with and without singletons at a 10% divergence threshold resulted in a single OTU for each individual.

#### Untagged individuals community

The Untagged Community contained individuals of 61 species, but only 49 of these (80%) were confirmed to have been successfully amplified and sequenced (Table S3). These reads generated 57 OTUs (82 OTUs with singleton sequences included) when clustering with UPARSE at the 3% threshold level (Table S6). Of these 57 OTUs, five did not return a hit when BLASTed against our local database. When these sequences were BLASTed against the NCBI-nt database, they were identified as either “uncultured eukaryote” or species not thought to be contained within the community (*Lecithaster gibbosus* and *Pleuroxus aduncus*). It is possible that these OTUs represent artifactual or contaminant sequences. On the other hand, *Ceriodaphnia lacustris* was not detected among our raw reads, but a cladoceran species clearly generated three OTUs, so it is also possible that this individual was misidentified and was in fact *Pleuroxus aduncus* or another chydorid.

The 52 OTUs that generated a local BLAST hit matched 42 species (of 49 successfully amplified and sequenced). Although we included a reference sequence within our BLAST database for each species within the community, some of the sequences from closely related species differed by <3% divergence, making it difficult to resolve them. For example, both *Artemia salina* and *A. franciscana* were included in the community and successfully sequenced as determined from the raw reads, but only *A. salina* was identified among the OTUs at the 3% threshold. Indeed, the reference sequences for these two species differ by only 0.25%. *Balanus crenatus* and *B. glandula*, two closely related species whose reference sequences differ by 3%, were both detected in the raw reads, but only one was identified via OTUs at the 3% threshold. While three *Daphnia* species were successfully amplified and pyrosequenced, the three OTUs generated identified only two *Daphnia* species, *Daphnia parvula* and *Daphnia pulex*. The reference sequence of the latter is just 1% divergent from the reference sequences of the third species, *Daphnia pulicaria*. Similarly, three OTUs matching *Gammarus* were generated, but only two of the three *Gammarus* species sequenced were identified by these OTUs. Thus, it is clear that some closely related species will go unidentified because they will cluster together into a single OTU when a divergence threshold of 3% is used. Clustering the data without singletons using a 1% threshold did not result in detection of additional *Balanus, Daphnia,* or *Gammarus* species.

A number of individuals (*Corbicula fluminea, Diaphanosoma brachyurum*, *Euytemora affinis, Leptodora kindtii*, *Macrocyclops albidus*, *Pseudocalanus mimus*) generated two OTUs at the 3% threshold when singletons were excluded. The OTUs generated by each of these individuals were not represented by equal numbers of sequences, with the exception of *Leptodora kindtii* where the ratio of sequences making up each OTU was 47: 43. The ratio for the other individuals ranged from ∽99.9: 0.1 to 80: 20.

When singletons were included, 25 extra OTUs were generated, but only two species from the community that were previously not found were now detected (*Balanus crenatus* and *Chthamallus dalli*) (Table S6). The number of OTUs that did not generate a hit when BLASTed against our local database also increased from five to 14 when singletons were included. When BLASTed against the NCBI-nt database, seven of these matched nontarget species, such as cercozoans and algae that were possibly found attached to or inside of the target species. Thus, it seems that including singletons in the cluster analysis often complicates the correlation of OTU number with species richness.

**Figure 2:**
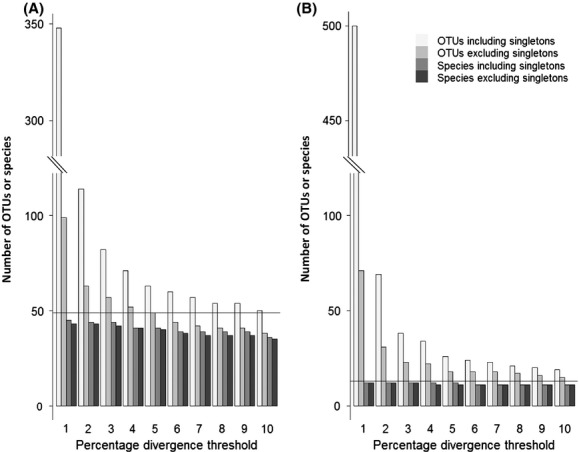
The number of OTUs generated and species detected when clustering data from (A) the Untagged Individuals Community and (B) the Untagged Populations Community. Filtered sequences were clustered into OTUs that were BLASTed against a reference database to assign species names. The solid horizontal line indicates the expected number of species. Percent divergence thresholds between 1% and 10% were used to cluster unique sequences with UPARSE, with and without including singletons in the analysis.

While clustering at a divergence threshold lower than 3% (i.e., 1% or 2%) resulted in the generation of many more OTUs, only one additional species was recovered by these OTUs (*Chthamalus dalli* when singletons were excluded and *Limnocalanus macrurus* when singletons were included; Fig.2A), suggesting that the 3% threshold is an appropriate level for most of the species in our datasets.

### Populations community

A total of 625,239 raw sequences were generated for the pooled Tagged and Untagged Populations Communities. Filtered sequences were subsequently separated into two datasets corresponding to the Tagged and Untagged Communities, encompassing 404,052 and 199,871 sequences, respectively, after removal of barcode and primer errors (Table1).

#### Tagged populations community

Tagged populations consisted of 24 groups of conspecific individuals, with the number of individuals classified as low, medium, or high. The sampling location was always identical within a population, but in some cases, the different populations (e.g., low vs. high) originated from different geographic locations (Table S2). Sequences generated for each tagged population were analyzed separately to avoid clustering of sequences across populations; we analyzed each dataset as a distinct population to avoid influencing the outcome of OTU clustering due to the presence of alleles specific to certain populations. Fifteen of the 24 populations (63%) generated a single OTU when singletons were excluded. While a number of populations generated multiple OTUs, we did not find evidence for a trend of increased number of OTUs with increased population size (Pearson's product moment correlation coefficient: *r* = 0.197, *P* = 0.357, singletons excluded; *r* = 0.147, *P* = 0.494, singletons included) (Table3). In some cases (e.g., *Daphnia* spp.), the number of reads contributing to OTUs increased with population size, even though no additional OTUs were generated with more individuals. We pooled the populations such that each individual would contribute an equal amount of DNA. Thus, it was expected that larger populations would generate more reads, as was the case (*r* = 0.605, *P* = 0.002, singletons excluded; *r* = 0.626, *P* = 0.001, singletons included). We tested the possibility that a greater number of reads resulted in the generation of more OTUs, but found no evidence for a correlation between the number of filtered reads clustered per population and the number of OTUs produced (Pearson's correlation: *r* = 0.051, *P* = 0.814, singletons excluded; *r* = −0.004, *P* = 0.982 singletons included).

**Table 3:** OTUs generated after clustering the data for the Tagged Populations Community using a 3% divergence threshold. Results are reported when singletons are excluded or included. The number of individuals included within each population is indicated before the species name. For example, “5 x” indicates that five individuals were present. The number of filtered reads that were clustered to form each OTU is reported.

Tagged population	Singletons excluded			Singletons included		
	No. OTUs	Species matching OTU(s)	No. reads in OTU	No. OTUs	Species matching OTU(s)	No. reads in OTU
5 × *Corbicula fluminea*	2	*Corbicula fluminea*	14,849	3	*Corbicula fluminea*	15,197
		*Corbicula fluminea*	11		*Corbicula fluminea*	12
					*Corbicula fluminea*	2
10 × *Corbicula fluminea*	1	*Corbicula fluminea*	1465	3	*Corbicula fluminea*	1735
					*Corbicula fluminea*	3
					*Corbicula fluminea*	1
30 × *Corbicula fluminea*	5	*Corbicula fluminea*	14,922	5	*Corbicula fluminea*	17,334
		*Corbicula fluminea*	36		*Corbicula fluminea*	50
		*Corbicula fluminea*	30		*Corbicula fluminea*	36
		*Corbicula fluminea*	9		*Corbicula fluminea*	12
		*Corbicula fluminea*	2		*Corbicula fluminea*	4
3 × *Neotrypaea californiensis*	1	*Neotrypaea californiensis*	4186	1	*Neotrypaea californiensis*	4793
5 × *Balanus crenatus*	2	*Balanus crenatus*	2294	2	*Balanus crenatus*	2863
		*Balanus glandula*	503		*Balanus glandula*	746
10 × *Balanus crenatus*	2	*Balanus crenatus*	19,560	2	*Balanus crenatus*	20,842
		*Balanus glandula*	2827		*Balanus glandula*	2963
17 × *Balanus* spp.	1	*Balanus crenatus*	8888	1	*Balanus crenatus*	9765
5 × *Crangonyx*	1	*Crangonyx spp*.	2326	1	*Crangonyx spp*.	2631
10 × *Hyalella clade 8*	3	*Hyalella azteca*	1681	3	*Hyalella azteca*	2164
		*Hyalella azteca*	603		*Hyalella azteca*	643
		*Hyalella azteca*	54		*Hyalella azteca*	71
5 × *Daphnia mendotae*	1	*Daphnia mendotae*	3901	2	*Daphnia mendotae*	4454
					*Daphnia pulex*	1
10 × *Daphnia pulex*	1	*Daphnia pulex*	12,825	1	*Daphnia pulex*	13,394
31 × *Daphnia pulex*	1	*Daphnia pulex*	30,041	1	*Daphnia pulex*	31,495
5 × *Leptodiaptomus minutus*	2	*Leptodiaptomus sicilis*	12,104	4	*Leptodiaptomus sicilis*	12,901
		*Leptodiaptomus sicilis*	4		*Leptodiaptomus sicilis*	8
					*Leptodiaptomus sicilis*	8
					*Leptodiaptomus sicilis*	1
9 × *Leptodiaptomus sicilis*	2	*Leptodiaptomus sicilis*	3697	3	*Leptodiaptomus sicilis*	2884
		*Leptodiaptomus sicilis*	10		*Leptodiaptomus sicilis*	2190
					*Leptodiaptomus sicilis*	14
30 × *Leptodiaptomus minutus*	1	*Leptodiaptomus sicilis*	38,038	2	*Leptodiaptomus sicilis*	39,947
					*Leptodiaptomus sicilis*	1
5 × *Diacyclops thomasi*	1	*Diacyclops bicuspidatus*	565	1	*Diacyclops bicuspidatus*	860
8 × *Diacyclops thomasi*	1	*Diacyclops bicuspidatus*	1343	1	*Diacyclops bicuspidatus*	1832
27 × *Diacyclops thomasi*	1	*Diacyclops bicuspidatus*	26,959	1	*Diacyclops bicuspidatus*	28,075
5 × *Leptodora kindtii*	1	*Leptodora kindtii*	10,915	2	*Leptodora kindtii*	6564
					*Leptodora kindtii*	4870
10 × *Leptodora kindtii*	1	*Leptodora kindtii*	2807	3	*Leptodora kindtii*	3441
					*Leptodora kindtii*	93
					*Leptodora kindtii*	1
28 × *Leptodora kindtii*	2	*Leptodora kindtii*	10,310	4	*Leptodora kindtii*	12,246
		*Leptodora kindtii*	13		*Leptodora kindtii*	117
					*Leptodora kindtii*	73
					*Leptodora kindtii*	19
5 × *Limnoperna fortunei*	1	*Limnoperna fortunei*	3048	2	*Limnoperna fortunei*	3387
					*Limnoperna fortunei*	1
10 × *Limnoperna fortunei*	2	*Limnoperna fortunei*	15,569	3	*Limnoperna fortunei*	16,088
		*Limnoperna fortunei*	445		*Limnoperna fortunei*	577
					*Limnoperna fortunei*	6
30 × *Limnoperna fortunei*	1	*Limnoperna fortunei*	3924	2	*Limnoperna fortunei*	4593
					*Limnoperna fortunei*	2

**Figure 3:**
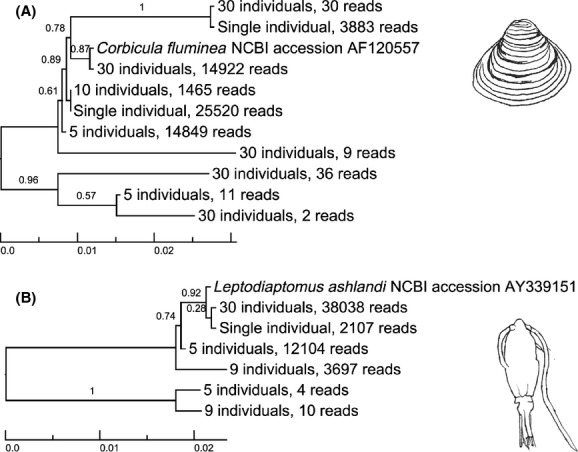
Dendrograms of OTU sequences from the Tagged Individuals and Tagged Populations Communities in (A) *Corbicula fluminea* and (B) *Leptodiaptomus* spp. The divergence threshold used was 3% and singletons were excluded. Representative sequences for the OTUs and a reference sequence were aligned with default settings in MAFFT v 7.150b (Katoh and Standley 2013). Dendrograms were generated using FastTree v 2.1.7 (Price et al. 2010). Each OTU was labeled according to the number of individuals included in the tagged sample (e.g., five individuals) and the number of reads that make up the OTU cluster (e.g., 14,849 reads).

When singletons were included, only eight of the 24 (33%) populations generated a single OTU when clustered at a 3% threshold. The maximum number of OTUs generated per population was five. The divergence threshold necessary to produce a single OTU for each population differed quite substantially in some cases and depended on whether singletons were included or excluded. Higher divergence thresholds were often necessary to produce a single OTU when singletons were included (Table4). When examining different population sizes of the same species, little consistency was observed in the divergence thresholds necessary to generate a single OTU, with the exception of *Diacyclops thomasi* and *Daphnia* spp., for which all populations generated a single OTU at each divergence threshold >1% when singletons were excluded. For example, 5, 10, and 30 individuals of *Corbicula fluminea* generated 2, 1, and 5 OTUs, respectively, but 5, 9, and 30 individuals of *Leptodiaptomus* spp. generated 2, 2, and 1 OTUs. We aligned the representative sequences of the OTUs generated for the *Corbicula fluminea* and *Leptodiaptomus* spp. populations and found that OTUs that are highly divergent within populations are also found across populations (Fig.3), suggesting that they could represent true biological variants.

**Table 4:** Lowest percentage divergence thresholds required to generate a single OTU when clustering data for the 24 populations in the Tagged Populations Community. The number of individuals included within each population is indicated before the species name. For example, “5 x” indicates that five individuals were present. Note that >10% indicates that multiple OTUs were still generated even when applying a 10% divergence threshold.

	Percentage identity required to generate a single OTU	
	Singletons excluded	Singletons included
5 × *Corbicula fluminea*	8	8
10 × *Corbicula fluminea*	3	8
30 × *Corbicula fluminea*	9	9
3 × *Neotrypaea californiensis*	2	3
5 × *Balanus crenatus*	4	6
10 × *Balanus crenatus*	4	4
17 × *Balanus* spp.	3	3
5 × *Crangonyx*	1	2
10 × *Hyalella clade 8*	5	6
5 × *Daphnia mendotae*	2	>10
10 × *Daphnia pulex*	2	3
31 × *Daphnia pulex*	2	3
5 × *Leptodiaptomus minutus*	4	4
9 × *Leptodiaptomus sicilis*	5	5
30 × *Leptodiaptomus minutus*	3	10
5 × *Diacyclops thomasi*	1	2
8 × *Diacyclops thomasi*	2	3
27 × *Diacyclops thomasi*	2	3
5 × *Leptodora kindtii*	3	4
10 × *Leptodora kindtii*	2	>10
28 × *Leptodora kindtii*	5	5
5 × *Limnoperna fortunei*	2	5
10 × *Limnoperna fortunei*	6	>10
30 × *Limnoperna fortunei*	2	4

#### Untagged populations community

Of the 14 species included in the Untagged Populations Community, 13 (93%) were successfully amplified and sequenced (Table S7). These reads generated 23 OTUs at the default threshold of 3%, or 38 OTUs with singleton sequences included. Of the 23 OTUs generated without singletons, five did not generate BLAST hits with our local database, but when examined more closely, one of these generated a hit that was too short or of insufficient percentage identity to be classified as a good match. The 18 remaining OTUs matched 12 of the 13 (92%) species contained in the assemblage. Four species were represented by multiple OTUs when singletons were excluded (*Artemia* spp., *Corbicula fluminea*, *Hyalella*, and *Paleamonetes* spp.). With singletons included, 38 OTUs were generated and 27 of these generated BLAST hits with our local database. The additional 9 OTUs generated with singletons included matched a species already detected when singletons were excluded, and no additional species were recovered. Clustering both the singletons excluded and included datasets at divergence thresholds lower than 3% (i.e., 1% and 2%) did not result in the identification of any additional species (Fig.2B). Thus, although the number of OTUs generated exceeds the number of species included within the community, the 3% threshold was sufficient to identify all species.

## Discussion

A central analytical task in metabarcoding studies is to classify sequences as entities or OTUs that correspond to species, a process of sequence clustering sometimes termed OTU picking (Sun et al. 2012). OTU-based methods are advantageous in that they allow assignment of sequences to OTUs even when reference taxonomic information is not available. A number of different OTU clustering algorithms exist that permit exploration of the genetic diversity within communities, some of which apply more flexible divergence thresholds than the algorithm we applied here (Box 1). Regardless of how these various OTU clustering algorithms structure genetic diversity, when OTU clustering is used to estimate species richness, the relationship between genetic diversity and Linnaean species should be understood. Through the use of mock communities, some of which were amplified with tagged primers, we demonstrate a means by which to assess intra- and interspecific diversity and to examine the correspondence between OTU number and expected species. While we used 454 pyrosequencing, rather than Illumina sequencing, the method we propose is not specific to any sequencing technology.

**Box1 Approaches to dealing with inaccurate estimation of biodiversity due to the application of a single divergence threshold across divergent taxonomic groups when OTU clustering**

**General Considerations**

Artificially assembled or mock communities with known numbers of species have previously been used to validate species richness estimates generated by metabarcoding (e.g., Behnke et al. 2011; Ihrmark et al. 2012; Kermarrec et al. 2014). However, mock communities can also be used to (1) calibrate the sequence divergence threshold used to delineate species by providing group-specific thresholds and (2) evaluate the level of intra- and interspecific divergence to ensure that the former does not exceed the latter.

**Suggested Approaches**

**Experimental design**

**Amplify species with tagged primers**

Our results show that mock communities could be constructed using a nested design that involves tagged primers to allow exploration of various levels of biological organization (intragenomic, intraspecific, and interspecific). This versatile approach allows separation of the sequences generated by single individuals or populations of various species or taxonomic groups. Through this approach, it becomes feasible to determine whether certain species or groups are more often over- or underestimated in species richness estimates.

**Employ alternative metabarcode markers**

The use of a single divergence threshold for OTU clustering across diverse taxonomic groups might be more appropriate when using alternative markers, such as COI, that exhibit less extensive length or nucleotide variation than the hypervariable regions of rRNA genes.

**Classifying sequences (OTUs) into species**

**Use alternative clustering algorithms**

Algorithms that avoid the use of a single “hard” threshold (such as 3%) across an entire dataset could allow the use of different divergence thresholds for some taxonomic groups. For example, CROP (Hao et al. 2011) implements a “soft” threshold method designed to infer optimal clustering results based on the natural organization of the data without setting an equal divergence threshold for every cluster. Another algorithm, Swarm (Mahé et al. 2014), takes a similar approach by first clustering highly similar amplicons iteratively using a user-defined threshold and then using internal structure and amplicon abundances of a cluster to refine the results.

**Employ a phylogenetic approach to sort reads by major taxonomic group**

Sorting quality-filtered reads or OTUs generated using a predefined generally accepted threshold by higher taxonomic groups (Order or Family levels) would allow different divergence thresholds to be applied to different groups.

**Relate variation in rDNA sequences to secondary structure of rRNA**

Overlaying the rRNA secondary structure model on filtered sequences could allow researchers to distinguish between genuine biological variation and artifactual variation or pseudogenes that represent nonfunctional gene copies (e.g., sequences that violate the secondary structure). This approach could potentially be worked into OTU clustering protocols as an additional screening for artifactual sequences. Incorporating models of sequence evolution in clustering workflows could greatly reduce the intragenomic variation detected and improve clustering efficiency.

We show that following commonly accepted conventions (i.e., using a 3% divergence threshold for OTU clustering with a robust algorithm) to analyze relatively simple datasets (i.e., the Individuals Communities) generates a relatively good correspondence between species richness and OTU number in the majority of cases; 74–84% of tagged species (Table2) and 59–73% of untagged genera/families (Table S6) showed a 1:1 correspondence. However, when multiple individuals of the same species were present within a sample and singletons were included in the clustering analysis, only about one-third of the tagged species (Table3) and untagged genera/families (Table S7) generated a 1:1 correspondence between OTU number and the expected number of species. Our findings thus support previous work (e.g., Behnke et al. 2011) that demonstrated difficulty in using a single divergence threshold to define OTUs when examining complex communities consisting of phylogenetically divergent groups.

When analyzing the Tagged Individuals Community dataset, the 3% divergence threshold generated strong correspondence between OTU and species number, with 84% of individuals generating a single OTU when singletons were excluded and 74% when singletons were included (Table2). However, to generate a single OTU for all individuals, we had to use an unreasonably high divergence threshold (10%). Applying such a threshold when analyzing metabarcoding data from a natural, complex community would result in OTUs shared between closely related species, such that OTU number would underestimate species richness. When analyzing the Tagged Populations Community dataset, the 3% divergence threshold generated results that were less clear. While the majority (63%) of tagged populations generated a single OTU with singletons excluded (33% with singletons included), the number of OTUs generated from populations of the same species but of different sizes varied extensively (Table3). OTU number did not increase with population size, suggesting that sampling more individuals does not necessarily lead to increased levels of intraspecific variance. Our findings may instead represent an effect of random sampling of alleles across populations, which sometimes differ in origin and thus in demographic history. If levels of intra-individual variation are high, the inclusion by chance of an individual with a divergent genotype in a “smaller” population may result in the generation of more OTUs than would be generated from a larger population. For example, *Corbicula fluminea* demonstrated high levels of both intra-individual and within-population variation across our datasets. We also found that divergent OTUs were shared across *Corbicula* populations, suggesting that such variation is unlikely to be generated by sequence or PCR errors.

The need for such high percentage divergence thresholds to generate a single OTU for some species is also affected by the method employed by the UPARSE-OTU algorithm to calculate sequence divergence (Flynn et al. 2015; THIS ISSUE). The indel rate is known to be high in the V4 region of 18S, and if sequences are not aligned before they are trimmed to the same length, as was the case here, the presence of internal indels will result in terminal gaps between sequences of the same overall length. The UPARSE-OTU algorithm considers terminal gaps to be differences, and thus the inclusion of both an indel and a terminal gap when calculating sequence divergence may drive divergence above 3% and cause the generation of multiple OTUs at this threshold. The UPARSE manual clearly warns users about analyzing globally alignable sequences, with a recommendation to trim reads to a fixed length unless full-length amplicons of high quality that reach the reverse primer are retained. We chose to trim our reads at a fixed length because of the extensive length variation in the V4 region of eukaryotes, which can vary among species by hundreds of nucleotides (Choe et al. 1999; Giribet and Wheeler 2001; Wuyts et al. 2001; Milyutina et al. 2001). It is not possible to design V4 primers that will generate an amplicon of at least 400 bp that can be completely sequenced in all eukaryotic species. Thus, the most viable alternative is to generate a ∽400-bp amplicon in species with short V4 regions and then trim all the sequences to 400 bp.

We found that indels can generate high intraspecific sequence divergence (e.g., in the case of the tagged individual of *Palaemonetes* spp.), but base pair substitutions can also contribute to divergent OTUs (e.g., in the case of the tagged individual of *Corbicula fluminea* and the populations of *Corbicula* and *Leptodiaptomus* spp.). Further examination of the sequence variants present within our data and their impact on OTU clustering is currently underway. lt is difficult to distinguish between sequencing artifacts and intragenomic variation, and while pyrosequencing is known to have high error rates in homopolymer regions (which could introduce artificial gaps), the presence of indels that occur in nature will likely have an impact on OTU clustering in any study that examines length variable markers, such as the rRNA genes. For example, intraspecific length variation is not uncommon in the V4 region (Crease and Taylor 1998). A survey of the V7 region of 134 individual *Daphnia obtusa* from 33 ponds across the U.S.A. also revealed extensive intragenomic length variation (McTaggart and Crease 2009). Individuals contained up to six length variants, which differed from one another by as much as 14 bp. The average was 2.6 variants per individual. Here, we found that an individual *Leptodora* contained two alleles (OTUs) in nearly equal frequency (47:43), and these alleles differed by one transversion and 1-bp or 2-bp indels at five sites. We also identified a number of length variable alleles at lower relative frequencies. In their study of *D. obtusa,* McTaggart and Crease (2009) identified both common and rare variants. Although intragenomic length variation does not affect all taxa to the same extent, it will inflate biodiversity estimates in some groups if it is not taken into consideration.

Other types of sequence errors (i.e., those not involving indels) could be interpreted as representing unique haplotypes (Sogin et al. 2006), and may even drive sequence divergence from the most common haplotype over three percent and thus generate new OTUs, as witnessed by Decelle et al. (2014). As the number of PCR/sequencing errors per base position is expected to increase with the number of sequences generated (also referred to as sequencing depth) (Lindner et al. 2013), we assessed the relationship between OTU number and the postfiltered sequencing depth for tagged populations. We did not find a correlation, suggesting that at least some of the multiple OTUs generated by a single species represent genuine biological variants. This finding might lend support for the inclusion of singletons, which are often considered to be artifactual sequences, in OTU clustering analyses. Including singletons resulted in the generation of additional OTUs for some species that were already identified (Tables3, S6 and S7; Fig.2), but in a few of cases, it also allowed discovery of previously undetected species. This suggests that if species are present at low abundance within the sequence data, they may be identified when singletons are included in the analysis. While we aimed to equally represent each individual within our communities, rDNA copy number varies substantially between species (Prokopowick et al. 2003), and sequences from species with low rDNA copy number, low cell number per individual, and/or small body size may be underrepresented in the data. In such cases, it could be argued that retaining singletons may allow the detection of rare species. On the other hand, if the research goal is to conservatively estimate species richness based on the presence/absence rather than relative abundances, discarding singletons is strongly advised.

Overall, a maximum of five OTUs was generated from a single tagged individual or tagged population, and in some cases, even applying a divergence threshold of 10% did not generate a single OTU. The expectation of a 1:1 ratio between OTU number and species richness is therefore unrealistic, especially when working with taxonomically divergent groups and highly variable regions of rRNA genes, and when using sequence divergence calculations that treat terminal gaps as differences. Given such apparently high levels of sequence variation, a 3% dissimilarity threshold to define OTUs may result in overestimation of biodiversity if species are split into multiple OTUs, whether or not these OTUs represent genuine variants. However, applying higher thresholds (i.e., >3%) could, in some cases, result in merging of genera or even orders. Even at 3% we struggled to discriminate closely related pairs of species in the genera Artemia and Daphnia based on variation in V4 sequences. The species commonly referred to as “*Artemia salina”* consists of several closely related species or subspecies, with *Artemia franciscana* being the main North American species. Even though individuals of these two species were present in the community, as the raw reads suggest, they were collapsed into one OTU at a 3% divergence threshold, which is not surprising given the low divergence (<1%) between the sequences in our reference database. As with *Artemia, Daphnia pulex* and *D. pulicaria* are very closely related and could not be distinguished even with a 1% divergence threshold. Overall, clustering at a divergence threshold lower than 3% did not result in many more species being recovered, suggesting it may not be possible to distinguish very closely related sister species even at 1%. This finding might explain why the number of OTUs generated by both of the Untagged Communities exceeded the number of actual species, yet some species still went undetected.

### Future directions

Our findings suggest that often OTU numbers do not reflect species richness and that alternative approaches for analyzing metabarcoding data and classifying OTUs/species may be required (Box 1). Using mock communities and a hierarchical approach of tagging single individuals and populations, we were able to sort sequences taxonomically prior to OTU clustering (Fig.1). This approach greatly facilitates the ability to identify the most appropriate divergence thresholds for different species or taxonomic groups, which we have shown differs across groups of zooplankton. Such group-specific thresholds could be applied when analyzing complex natural communities. For example, sequences could be sorted taxonomically post-PCR using a combination of phylogenetic approaches that evaluate the phylogenetic relationship of OTUs and taxonomic assignment by BLASTing against comprehensive sequence databases. Reads sorted by broad taxonomic groups could then be clustered into OTUs using user-defined group-specific thresholds (Box 1). If an appropriate threshold is not known, a wide range of divergence threshold values could be explored. At each threshold, the resulting OTUs could be BLASTed against a comprehensive database to assess whether a 1:1 correspondence between OTU and Linnaean species is achieved. This approach makes the assumption that databases are well represented and that the marker used has a sufficient gap between intra- and interspecific divergences. Our results suggest that for a few species, intraspecific divergence likely exceeds interspecific divergence. This problem may not be restricted to the V4 region of the 18S. Artifactual sequences and pseudogenes are likely to generate large intra-individual variation that could often be interpreted as “rare” biodiversity.

With this in mind, we envision an additional filtering step that could be incorporated in order to remove sequences that disrupt the secondary structure of rRNA markers. For example, OTU sequences could be mapped against the secondary structure of rRNA, with the expectation that genuine substitutions or indels may be unlikely to occur in highly conserved regions and result in changes in the secondary structure. Decelle et al. (2014) inspected alignments of V4 sequences and found that most minor variants contained substitutions that seemed to be randomly distributed and were not preferentially located in the variability hotspot region of their reference sequences. They also found that the secondary structure of the minor variants was generally different from the dominant sequence, confirming that the substitutions were probably artifactual. Given the complexity of metabarcoding datasets, we suggest that future analysis should incorporate well-developed models of sequence evolution. The application of these models would allow researchers to remove nonfunctional sequences of either biological or artifactual origin and thus reduce the generation of spurious OTUs. Such a filtering approach has the potential to further diminish the level of intragenomic variation detected within high-throughput datasets and thus also widen the gap between intra- and interspecific variation.

## Conclusions

Metabarcoding holds particular promise where the potential for taxonomic identification of species is limited. However, we show that when using rRNA gene sequences to describe complex communities that cover a wide taxonomic range and consist of species present at varying densities, a single sequence divergence threshold does not always generate good correspondence between OTU number and species richness. We advocate sorting reads taxonomically prior to OTU clustering, and using a flexible divergence threshold. Issues related to the use of a uniform divergence threshold may be less extensive if alternative markers are applied that are less prone to length variation than the rRNA genes (Box 1). rDNA markers are often used for metabarcoding studies due to their high copy number, but this asset becomes disadvantageous if intragenomic variation creates a substantial number of spurious OTUs. The presence of indels within rRNA gene sequences may also cause problems during OTU clustering if gaps are not appropriately treated (Flynn et al. 2015; THIS ISSUE). Considering the nature of rRNA genes (McTaggart and Crease, 2005; McTaggart and Crease 2009; Nyaku et al. 2013), the issues raised here are likely to affect any study that applies these markers, regardless of the particular HTS technology employed. Given the conceptual and practical difficulty in translating OTUs to species, we argue that alternative approaches should also be considered when attempting to describe community composition.

## Data Accessibility

The 454 data generated in this study have been deposited in the Sequence Read Archive. Accession numbers are shown in Tables S1-S4. Sanger sequences are provided in Table S5.

## Supporting Information

**Figure S1.** Sequence quality (PHRED quality score) per base position for the Untagged Individuals Community dataset, as reported by FastQC (Andrews, 2010). **Table S1.** Individuals included in the Tagged Individuals Community. **Table S2.** Populations included in the Tagged Populations Community. **Table S3.** Species included in the Untagged Individuals Community (Sequence Read Archive accession number SRX884895). **Table S4.** Species included in the Untagged Populations Community (Sequence Read Archive accession number SRX884904). **Table S5.** V4 18S Sanger sequences generated in this study and included in our local BLAST databases. **Table S6.** The number of OTUs generated and species detected when clustering at 3% (with singletons either excluded or included) compared to the number of expected species, for each genus or family included in the Untagged Individuals Community. **Table S7.** Species detected, listed by Genus or Family, when clustering data generated by the Untagged Populations Community at 3% when singletons are either excluded or included.
