# Supplementary material for: Divergence thresholds and divergent biodiversity estimates: can metabarcoding reliably describe zooplankton communities?
Source: Ecol Evol. 2015 May 13;5(11):2234–51. doi: 10.1002/ece3.1485 (PMC4461424; doi:10.1002/ece3.1485)
Supplement: Supplementary file 1 [file ece30005-2234-sd1.docx]

**Supplementary information**

Table S1. Individuals included in the Tagged Individuals Community. The DNA of a single individual of each species was extracted and PCR amplified using tagged primers, with the exception of the rotifer *Brachionus calyciflorus*, for which 20 individuals of the same clone were extracted and PCR amplified due to their small size. Sequence Read Archive (SRA) accession numbers are provided.

| **Group** | **Species** | **Geographic origin** | **Provider** | **SRA accession** |
| --- | --- | --- | --- | --- |
| Amphipod | *Echinogammarus ischnus* | Lake Erie, Nanticoke, Ontario | Colin Van Overdijk | SRX895449 |
| Amphipod | *Themisto libellula* | Chukchi Sea, Alaska, USA | Adriana Radulovici | SRX895485 |
| Anostraca | *Artemia salina* | Unknown | Live Aquaria | SRX894166 |
| Cirripedia | *Balanus crenatus* | Puget Sound, USA | Hilary Hayford | SRX894167 |
| Cladocera | *Cercopagis pengoi* | Nanticoke, Ontario | Colin Van Overdijk | SRX894170 |
| Cladocera | *Daphnia mendotae* | Lake Erie, Nanticoke, Ontario | Colin Van Overdijk | SRX895396 |
| Cladocera | *Pleuroxus denticulatus* | Huntsville, Ontario | Colin Van Overdijk | SRX895483 |
| Copepoda (calanoid) | *Epischura lacustris* | Nanticoke, Ontario | Colin Van Overdijk | SRX895460 |
| Copepoda (calanoid) | *Leptodiaptomus ashlandi* | Lake Huron, Ontario | Colin Van Overdijk | SRX895465 |
| Copepoda (calanoid) | *Senecella calanoides* | Huntsville, Ontario | Colin Van Overdijk | SRX895484 |
| Copepoda (cyclopoid) | *Diacyclops thomasi* | Huntsville, Ontario | Colin Van Overdijk | SRX895455 |
| Copepoda (cyclopoid) | *Mesocyclops edax* | North Frontenac, Ontario | Colin Van Overdijk | SRX895467 |
| Copepoda (harpacticoid) | *Microsetella norvegica* | Steensby Inlet, Canada | Siobhan Curry | SRX895468 |
| Decapoda | *Cancer spp.* | Hawkesbury, Canada | Siobhan Curry | SRX894169 |
| Decapoda | *Palaemonetes spp*. | Unknown | Live Aquaria | SRX895470 |
| Mollusca | *Corbicula fluminea* | St Lawrence River, Quebec, Canada | Emilija Cvetanovska | SRX894171 |
| Mollusca | *Dreissena polymorpha* | Quebec City, Canada | Elizabeta Briski |  |
| Rotifera | *Brachionus calyciflorus* | Onandaga Lake, New York, USA | Guntram Weithoff | SRX894168 |
| Tunicate | *Ciona intestinalis* | Cardigan River, Prince Edward Island, Canda | Aibin Zhan | SRX894172 |
| Tunicate | *Oikopleura labradoriensis* | Foxe Basin, Steensby Inlet, Canada | Siobhan Curry | SRX895469 |

Table S2. Populations included in the Tagged Populations Community. The number of individuals of each species included in the population is listed in the table. Each of these populations was separately DNA extracted and PCR amplified using tagged primers. Sequence Read Archive (SRA) accession numbers are provided.

| **Group** | **Species** | ***N*** | **Geographic origin** | **Provider** | **SRA Accession** |
| --- | --- | --- | --- | --- | --- |
| Amphipod | *Crangonyx* | 5 | Unknown | Jonathan Witt | SRX895495 |
|  | *Hyalella clade 8* | 10 | Unknown | Jonathan Witt | SRX895679 |
| Cirripedia | *Balanus crenatus* | 5 | Puget Sound, USA | Hilary Hayford | SRX895488 |
|  | *Balanus crenatus* | 10 | Puget Sound, USA | Hilary Hayford | SRX895676 |
|  | *Balanus* spp. | 17 | Nanticoke, Canada | Siobhan Curry | SRX895682 |
| Copepoda (calanoid) | *Leptodiaptomus sicilis* | 5 | Lake Huron, Ontario | Colin Van Overdijk | SRX895508 |
|  | *Leptodiaptomus sicilis* | 9 | Picton Ontario, Bay of Quinte | Colin Van Overdijk | SRX895765 |
|  | *Leptodiaptomus minutus* | 30 | Port Elgin, Ontario, Lake Huron | Colin Van Overdijk | SRX895690 |
| Copepoda (cyclopoid) | *Diacyclops thomasi* | 5 | Lake Ontario, Burlington Ontario | Colin Van Overdijk | SRX895501 |
|  | *Diacyclops thomasi* | 8 | Port Elgin, Lake Huron, Ontario | Colin Van Overdijk | SRX895674 |
|  | *Diacyclops thomas* | 27 | Kashwakamak Lake, North Frontenac, Ontario | Colin Van Overdijk | SRX895683 |
| Cladocera | *Daphnia mendotae* | 5 | Lake Erie, Nanticoke, Ontario | Colin Van Overdijk | SRX895498 |
|  | *Daphnia pulex* | 10 | Lake Simon, Ontario | Katie Millette | SRX895678 |
|  | *Daphnia pulex* | 31 | Lake Kelly, Ontario | Katie Millette | SRX895691 |
| Cladocera | *Leptodora kindtii* | 5 | Nanticoke, Lake Erie, Ontario | Colin Van Overdijk | SRX895517 |
|  | *Leptodora kindtii* | 10 | Huntsville, Peninsula Lake, Ontario | Colin Van Overdijk | SRX895680 |
|  | *Leptodora kindtii* | 28 | ORO Station, Lake Simcoe,  Ontario | Colin Van Overdijk | SRX895685 |
| Decapoda | *Neotrypaea californiensis* | 3 | Vancouver, Canada | Siobhan Curry | SRX895487 |
| Mollusca | *Limnoperna fortunei* | 5 | Argentina | Sara Ghabooli | SRX895670 |
|  | *Limnoperna fortunei* | 10 | Argentina | Sara Ghabooli | SRX895681 |
|  | *Limnoperna fortunei* | 30 | Argentina | Sara Ghabooli | SRX895688 |
| Mollusca | *Corbicula fluminea* | 5 | St Lawrence River, Canada | Emilija Cvetanovska | SRX895492 |
|  | *Corbicula fluminea* | 10 | Upper Clinch River, USA | Emilija Cvetanovska | SRX895677 |
|  | *Corbicula fluminea* | 30 | Lake George, USA | Emilija Cvetanovska | SRX895686 |

Table S3. Species included in the Untagged Individuals Community (Sequence Read Archive accession number SRX884895). Each species was represented in the community as a single individual. In total, 61 species were included in the community, although only 49 were found to be successfully amplified and pyrosequenced. Species that were not found to be present among the raw unfiltered reads are highlighted in grey.

| **Group** | **Species** | **Geographic origin** | | **Provider** | |
| --- | --- | --- | --- | --- | --- |
| Amphipoda | *Crangonyx spp.* | Unknown | | Jonathan Witt | |
| Amphipoda | *Gammarus lacustris* | Unknown | | Jonathan Witt | |
| Amphipoda | *Gammarus lawrencianus* | St. Andrews, New Brunswick, Canada | | Adriana Radulovici | |
| Amphipoda | *Gammarus oceanicus* | Terra Nova, Newfoundland and Labrador, Canada | | Adriana Radulovici | |
| Amphipoda | *Hyalella azteca* | Unknown | | Jennifer Adams | |
| Amphipoda | *Hyalella* clade 1 | Unknown | | Jonathan Witt | |
| Amphipoda | *Hyalella* clade 8 | Unknown | | Jonathan Witt | |
| Amphipoda | *Hyperia galba* | Resolute, Nunavut, Canada | | Adriana Radulovici | |
| Amphipoda | *Hyperoche medusarum* | Resolute, Nunavut, Canada | | Adriana Radulovici | |
| Amphipoda | *Themisto libellula* | Chukchi Sea, Alaska, USA | | Adriana Radulovici | |
| Anostraca | *Artemia franscicana* | Unknown | | Jennifer Adams | |
| Anostraca | *Artemia salina* | Unknown | | Live Aquaria | |
| Cirripedia | *Balanus crenatus* | Puget Sound, USA | | Hilary Hayford | |
| Cirripedia | *Balanus glandula* | Puget Sound, USA | | Hilary Hayford | |
| Cirripedia | *Chthamalus dalli* | Puget Sound, USA | | Hilary Hayford | |
| Cladocera | *Bosmina longirostris* | Lake Erie, Nanticoke, Canada | | Colin Van Overdijk | |
| Cladocera | *Bythotrephes longimanus* | Huntsville, Canada | | Colin Van Overdijk | |
| Cladocera | *Ceriodaphnia lacustris* | Nanticoke, Canada | | Colin Van Overdijk | |
| Cladocera | *Daphnia obtusa* | Pallanza, Piemonte, Italy | | Alessandra Loria | |
| Cladocera | *Daphnia parvula* | Huntsville, Canada | | Colin Van Overdijk | |
| Cladocera | *Daphnia pulex* | Champaign- Urbana, USA | | Tiffany Chin | |
| Cladocera | *Daphnia pulicaria* | Champaign- Urbana, USA | | Tiffany Chin | |
| Cladocera | *Diaphanosoma brachyurum* | Nanticoke, Canada | | Colin Van Overdijk | |
| Cladocera | *Holopedium gibberum* | Huntsville, Canada | | Colin Van Overdijk | |
| Cladocera | *Leptodora kindti* | Lake Erie, Nanticoke, Canada | | Colin Van Overdijk | |
| Cladocera | *Polyphemus pediculus* | Lake Huron, Canada | | Colin Van Overdijk | |
| Copepoda (calanoid) | *Leptodiaptomus minutus* | Lake Huron, Canada | | Colin Van Overdijk | |
| Copepoda (calanoid) | *Limnocalanus macrurus* | Lake Erie, Nanticoke, Canada | | Colin Van Overdijk | |
| Copepoda (calanoid) | *Acartia tonsa* | Hawkesbury, Canada | | Siobhan Curry | |
| Copepoda (calanoid) | *Calanus finmarchicus* | Frobisher Bay Iqaluit, Canada | | Rob Young | |
| Copepoda (calanoid) | *Centropages abdominalis* | Burrard Inlet Vancouver, Canada | | Rob Young | |
| Copepoda (calanoid) | *Eurytemora affinis* | Quebec City, Canada | | Elizabeta Briski | |
| Copepoda (calanoid) | *Microcallanus pusillus* | Strait of Canso Hawkesbury,Canada | | Rob Young | |
| Copepoda (calanoid) | *Pseudocalanus mimus* | Nanticoke, Canada | | Siobhan Curry | |
| Copepoda (cyclopoid) | *Acanthocyclops vernalis* | Thunder Bay, Canada | | Siobhan Curry | |
| Copepoda (cyclopoid) | *Corycaeus anglicus* | Strait of Georgia Robert's Bank, Canada | | Rob Young | |
| Copepoda (cyclopoid) | *Eucyclops speratus* | Lake Erie, Nanticoke, Ontario | | Colin Van Overdijk | |
| Copepoda (cyclopoid) | *Macrocyclops albidus* | North Frontenac, Canada | | Colin Van Overdijk | |
| Copepoda (cyclopoid) | *Oithona atlantica* | Burrard Inlet Vancouver, Canada | | Rob Young | |
| Copepoda (harpacticoid) | *Clytemnestra scutellata* | Victoria, Canada | | Siobhan Curry | |
| Copepoda (harpacticoid) | Tachidiidae | Iqaluit, Canada | | Siobhan Curry | |
| Copepoda (harpacticoid) | *Tisbe furcata* | Hudson Strait Deception Bay, Canada | | Rob Young | |
| Copepoda (harpacticoid) | *Zaus abbreviatus* | Hudson Strait Deception Bay, Canada | | Rob Young | |
| Decapoda | *Carcinus maenas* | Strait of Canso Hawkesbury, Canada | | Rob Young | |
| Decapoda | Caridea | Deception Bay, Canada | | Siobhan Curry | |
| Decapoda | Crangonidae | Nanticoke, Canada | | Siobhan Curry | |
| Decapoda | Grapsidae | Vancouver, Canada | | Siobhan Curry | |
| Decapoda | Hippolytidae | Nanticoke, Canada | | Siobhan Curry | |
| Decapoda | Majidae | Frobisher Bay Iqaluit, Canada | | Rob Young | |
| Decapoda | *Neotrypaea californiensis* | Nanticoke, Canada | | Siobhan Curry | |
| Decapoda | Xanthidae | Strait of Georgia Nanaimo, Canada | | Rob Young | |
| Mollusca | *Corbicula fluminea* | Saint Lawrence River, Quebec, Canada | | Emilija Cvetanovska | |
| Mollusca | *Dreissena polymorpha* Quebec City, Canada | | | Elizabeta Briski | |
| Mollusca | *Limacina helicina* | Hudson Strait, Deception Bay, Canada | | Rob Young | |
| Mollusca | *Limnoperna fortunei* | Argentina | | Sara Ghabooli | |
| Mollusca | *Mytilus edulis* | Halifax, Canada | | Farrah Chan | |
| Mollusca | *Nassarius distortus* | Unknown | | Live Aquaria | |
| Mollusca | *Nerita spp.* | Unkown | | Live Aquaria | |
| Mollusca | Pteropoda | Unknown | | Siobhan Curry | |
| Tunicate | *Ciona intestinalis* | Cardigan River, Prince Edward Island, Canada | Aibin Zhan | |  |
| Tunicate | *Oikopleura labradoriensis* | Foxe Basin, Steensby Inlet, Canada | | Siobhan Curry | |

Table S4. Species included in the Untagged Populations Community (Sequence Read Archive accession number SRX884904). In total, 14 species were included in the community, and the number of individuals included in the community per species is listed in the table. Altogether 76 individuals were included in the community. Thirteen of the species included in the community were found to have been successfully amplified and sequenced; the species found to be missing from the raw unfiltered reads are highlighted in grey.

| **Group** | **Species** | ***N*** | **Geographic origin** | **Provider** |
| --- | --- | --- | --- | --- |
| Amphipod | *Gammarus lawrencianus* | 1 | St Andrews, New Brunswick, Canada | Adriana Radulovici |
| Anostraca | *Artemia* spp. | 2 | Unknown | Live Aquaria |
| Cirripedia | *Balanus crenatus* | 10 | Puget Sound, USA | Hilary Hayford |
| Cirripedia | *Chthamalus dalli* | 1 | Puget Sound, USA | Hilary Hayford |
| Cirripedia | *Hyallela clade 8* | 5 | Unknown | Jonathan Witt |
| Cladocera | *Leptodora kindtii* | 5 | Lake Erie, Nanticoke, Ontario | Colin Van Overdijk |
| Cladocera | *Daphnia pulex* | 10 | Canard Lake, Ontario, Canada | Genelle Harrison |
| Copepoda (calanoid) | *Leptodiaptomis minutus* | 1 | Lake Huron, Port Elgin, Ontario | Colin Van Overdijk |
| Copepoda (calanoid) | *Acartia longiremis* | 5 | Vancouver, Canada | Siohban Curry |
| Copepoda (calanoid) | *Eurytemora affinis* | 23 | Lake Erie, Nanticoke Ontario | Colin Van Overdijk |
| Decapoda | *Palaemonetes* spp. | 5 | Unknown | Live Aquaria |
| Decapoda | *Carcinus maenus* | 2 | Hawkesbury, Ontario | Siobhan Curry |
| Mollusca | *Corbicula fluminea* | 5 | Saint Laurence River, Quebec, Canada | Emilija Cvetanovska |
| Mollusca | *Nerita spp.* | 1 | Unknown | Live Aquaria |

Table S5. V4 18S Sanger sequences generated in this study and included in our local BLAST databases. Cloning was not used prior to sequencing, and the Ns generated may represent intra-individual length variation.

| >*Cancer spp.*  AAATCCTCATAGCGTAATTAAAGNTTGTTTGCGGTTTAAAAGCTCGTAGTTGGATTTCAGTTCTGGACTGACGGGTTCACCGCCCGGGTGCATACTGGTC  ACGCTCCGAACAGCCACAACAGCCCGCTGGCTCGCACGGGGTGCTCTTCATCGAGTGTCCCGCGTGGCCGGCAGAGTTTACTTTGAAAAAATTAGAGTGC  TCAAAGCAGGCTACACTGACGGCCTGAATGCCTAAGCATGGAATAATGGAATAGGACCTCGGTTCTATTTTGTCGGTTTTCTGAACCCGAGGTAATGACT  AATAGGAACAGGCGGGGGCATTCGTATTGCGACGCTAGAGGTGAAATTCTTGGACCGTCGCAAGACGAACTACTGCGAAAGCATTTGCCAAGGATGTTTTCATTAATCAAGAACGAAAGTTAGAGGTTCGAAGACGATCAGATACCGCCA  >*Chthamalus dalli*  NAAAATCCCNCGTNACGTAATTAAAGCTGGTTGCGGTTAAAAAGCTCGTAGTTGGATATCAGTGCGTGTCCGGTCCGGTATGCCCGGCGGCGGCGCTCGCGCGTCACTGCTGGGCTCCCAAATATCGGCTGGCCGCATTCAATCGTGCCGGATCCGTCGACGGGCCGTTCTTCGGAGGGGCCTGTTGGCGACCGGCGACGTTACCTTGAACAAATTAGAGTGCTCAAAGCAGGCTCTGAATGCCTGTATACATATTCATGGAATTGGAGAATACGTCCCTGGCTCGATTTGGTTGGTTTT  GAGAGTCGAAGGGAAATGATTAATAGGGACTGACGGAGGCATTCGTATTGCGACGCGAGGGGTGAAATCCTGTGACCGTCGCACGACGAACTACTGCGAAAGCATTTGCCGAGAATGTTTTCATTAGTCAAGAACGAAAGTTAGAGGTTCGAAGACGATCAGATACCGNCANA  >*Crangonyx spp.*  ATCCCTTNTNGCATCTATTAAAGTTTGCTGCGGTTTAAAANGCTCGCAGTTTGAATGTCAGTGCCAAGCGCAGGTTCAGGTGGACGCTGGGGTGTGAGAA  CCAACACGTGTACGGACGTCCTTTCGGAGCTAATGCCGAGACTGGACGCACCGGCCGTGTCTCACAAACTCCCGTCCGTGACAAGCCTCCTCGCTTGCGT  ACATATCGGAACTGGTTTTCGGAGGCTGCGAAAAGCCGGTGGTTTCAACCACGTCTTGCCAGTACGGGGCCTTCGGGTCGCCAACGCAGGCTTGACTGGG  GGTTGCCAGCTCGGTTCGCATGTCCTCTTTGTGGTTTCAGAGTCACCTTGAGTAAATCAGAGTGCTCAAAGCAGTCGTAAGGGCATTGCGCCCCATGAAA  AGTCTTGACGGACTGACTGGTGATGCATGGCATGTCGGAAGACGACGTCGCGTGCTGGCTCAGAGTTGAATCAGTGGTATAAGCCAGCCGCGACGTACTGATTAAAAGGGACGGTCGAGGGCATTGGTATTGCGAGGCGAGAGGTGAAATTCTATGACCCCCGCAAGACCTCCTGAAGCGAACGCGTCTGCCAAGGACGTTTTCATTAATCATGAACGAAAGTTANAGGATCGAAGACGATCAGATACCGCCAAANA  >*Daphnia parvula*  AANAAACGCTNAAGCGTAATAAAGTTGTTGCGGTAAAAAGCTCGTAGTCGGATGTCTGTCTTCGGCCGGGCGGCGCCGCTCTGAATCAAGGGTGTTGCGTTTCACGCTCTGGGAGCCCGGGTGTCAAAGCTCGACTCTCTTCACGGTCGGACAACACAGCCGGAGTGGATGTGGGTGAGTCGCGGTTGTATCGTTTTGAGAGTTCGTTCATGTGGGGTGTGTTTCGCGAGTGCGTGAAGCTGGTTAACGCCGGCCTTTGCGTTCGCGCGTCGTGCCGTCGTGTGCGTCTTTTATCTCGTTGACTCGATTTGCTCCTCCACAGTCAATCGTTCGGGGTGCCCTTCACCGGGTGTCTCGGGCGGCCGGCAACGTTTACTTTGAACAAATTAGAGTGCTCAAAGCAGGTGTATCCCAACGCCTGAATATCGCAGCATGGAATGATGGAATAGGACCTCGGTCCGATTTTGCTGGTCTTTTACTTTTGGACCCGAGGTAATGGTCAATAGAGACGGACGGGGGCATTCNTACTGCGGCGACAGAGGTGAAATTCTTGGACCGCCGCAAGACGAACAACAGCGAAGGCATTTGCCAAGAATGTTTTCCTTGATCAAGAACNAAAGTTAGAGGTTCGAAGACGATCANATACCGCCAAACANNTNCNNNNTNNACANCTCNNNNAAAAATAANANTNNAAAAAAAATAAAAAANNAATNNANNANANANAATATAAAANTNNAANNAANTAAAANANTAAANTANTANAAACCCAACAAANANATAAAANAAANACANNAANNACAAANANATAAAAAATAAATATACAANNAAAANACACTAAANAAANAAANNAAANAAAANAAATNANANAAANAATAAAANNATNNNNAAAAANNNNNTAAANNAANNCANANANAACAAANAANANAAACCTAACANAAAAAANNANANAACAANACNCAATANAAAAAAACAAAAANNNACCCAAAAANNNAANNNTAAATATTNACANTACAAANNATCATACAAANNATCNNCATANNANTATNACANNNNNNATANACACANANNANNTAAAAAANTNNNAAACANATACTCATANNAATANAAACNTATNNANNACCAAAAACAANNANNAAACAAAANAANATAANA  >*Daphnia mendotae*  GACAAMNSCTCANNSCGTANATTAAGNTTGTTTGCGGNTTAAAAGCTCGTAGTCNGATGTCTGTCTTCGGCCGGGCGGCGCCGCTCTGAATCAAGGGTGTTTGCGTTTCACGCTCTGGGAGCCCGGGTGTCAAAGCCCGACTCTCTTCACGGTCGGACAACATCGCCGGAGTGGTGGTCGGCGGGTCGCGGTTGTATCGGTTTCNATTCGGTTGAGTGCGAGAGTGNNTCNCNGGNNYCNCYCNTCNCNANCNGTNNYNNTNGNNTTGANNNSAWTCRCNNCACCCTANTCAAATCSTATNNGNGNGSCCNTNANCNGGNGKNYNRGGNNGNCNGMANCNTTTAMYTTNAANAAANTARARATGNNCMAARCNRGNGNAWCCCAANNNCNNNAWNNCNCAGCNTGGAANGRNGGAATANGACCNCNNNCCNAWATTTGNYGGWCNTTTNMTTTTGGANCCSARGNAANGGNCNATARARAMNATGAMNGGGGCNTTCNTAMNGNRGCNACMNARNTGAAAATTCNTGNNACNNTCCNCANRAMNAAMAANNANNNAARGNATTTGNCMANAATNKTTTNNTTNNNNCANRAACNAAANANNTARAGGTTCNAARAMNAWCARANAMACKNCANNNYMNNNNNNNNNNNNNNNNANNTMNMNNANNWRMNNNNNANNNNNNAANNWNNANAMANANNMAANANNNNNANNNANNNNNNNANNNMNNNCNNNANAANANNTNNNANNNNNAANWAWNNANAANNAMNNNNNNNNNNNNNNNNNNNANNNWANNAARNNNNANNNAWNANNNANNNNNNTCNNNANNNWNWMANNANNNANNNANANNMAWANNNANNANAWNANANNNANNAANNNANNNWAANNNANNNNNWMNANNNNAANWAANAANNNNNAANAMNNNWNNANANTANNNNAWAWAMNNAANMNANNNNNNANANMANANNYANNNMNTANNWWNNNANNNNNNNNNNNNANNNNNNANNNNNNNNNNANNYNNNTAANNNNNANNYAANANMNMNNANANNANNNNNNANNNMATTATNMANNANNNNNTNNANNNANNANNNNNNANANNAAYWNY  NNWNNNMNNANANNNNNNNNNNNNNNNMANNMAMNNCNNNNNNANNCACAACCNAACNNCATACNAATTNCANCTAAAANANANNAAGTNNNTCNNANANANNATNAANAANATAGCGAAANTCANTAACNNACANNNNA  >*Daphnia obtusa*  GNAANCCCTCAANCGTAATWAAGTTGTTGCGGTTAAAAAGCTCGTAGTCGGATGTCTGTCTTCGGCCGGGCGGCGCCGCTCTGAATCAAGGGTGTTGCGTTTCACGCTCTGGGAGCCCGGGTGTCAAAGCCCGACTCTCTTCACGGTCGGACAACATCGCCGGAGTGGTGGCCGGTGCGTCGTTGTTGTATCKGCGGTCMGAGTYTTGTTTGRAAATGTTCCTTCGGGTTCTWTTCTTTCNNAATTTTGGYCGMAKTTNACNTCRATTCNCTCCACCCAKTCAANCNTTCGGGGNGCCCTTCACCGGGNGNCNCGGGNGGCCGGNAACGTTWACTTTNAACAAATTARANNGCTCAAANCAGGNGNATCCCAACNCCNGAANATCNCASCANGRAANGANGNAANAGNACCNCGGTCCRATTTTGCNGGNCTTTNACTTTTGRACCCRAGGNAANGGTCAANARARACGNACGGGGGCATTCNNACNGCGGCRACARAGGTNAAATTCTTGRACCGCCNCAARACRAACAACANCNAAGGCATTTGCCAARAANGTTTTCCTTGATCAARAACRAAANTTARAGGTTCRAARACRATNARAATACCGNMA  >*Daphnia pulicaria*  NAAACCCTCAAAGCGTAATTAAGTTTGTTGCGGTTAAAAAGCTCGTAGTCGGATGTCTGTCTTCGGCCGGGCGGCGCCGCTCTGAATCAAGGGTGTTGCGTTTCACGCTCTGGGAGCCCGGGTGTCAAAGCCCGACTCTCTTCACGGTCGGACAACATCGCCGGAGTGGTGGTCGGTGCGTCGTTGTTGTATCTGCGGCCAGAGTTGGAAAGTTCTTTCGGGTTCTTTTTTAATTTTGGTCGTAGTTGACTCGATTCGCTCCACCCAGTCAATCGTTCGGGGTGCCCTTCACCGGGTGTCTCGGGCGGCCGGCAACGTTTACTTTGAACAAATTAGAGTGCTCAAAGCAGGTGTATCCCAACGCCTGAATATCGCAGCATGGAATGATGGAATAGGACCTCGGTCCGATTTTGCTGGTCTTTTACTTTTGGACCCGAGGTAATGGTCAATAGAGACGGACGGGGGCATTCGTACTGCGGCGACAGAGGTGAAATTCTTGGACCGCCGCAAGACGAACAACAGCGAAGGCATTTGCCAAGAATGTTTTCCTTGATCAAGAACGAAAGTTAGAGGTTCGAAGACGATCAGATACCGCCAANNN |
| --- |
| >*Epischura lacustris*  GGNAAATCGCTCATNGCGTAATTAAGGTTGGTTGTGGTTAAAAAGCTCGTAGTTGGATTTCGGCGGGTATTGGTCGGTTTGAATTGCTTCAATACTGACT  TTTTTACCCGTGTGTTTTGCCAGAATTTAACAGGTGATCTTCGCCGATTGTCCGTTAGAACTGGCAGGTTTACTTTGAAAAAATTAGAGTGCTCAAAGCA  AGCTTGATTGCTTGAATATTCGTGCATGGAATAATAGAATAGGAAGTCGTTTCTATTTTGTTGGTTTTCGGAAATCGACTTAATGATTAATAGGGATAGT  CGGGGGCATTTGTATTCAAACGACAGAGGTGAAATTCTTGGACCGTTTGAAGACAAACTACTGCGAAAGCATTTGCCAAGAATGTTTTCATTAATCAAGA  ACGAAAGTTAGAGGTTCGAAGACGATCAGATACCGCCANNNNNNNNNNNNNNNNNNNNNNNNANNCNNNANNANNNNTNNNNNCCCNNNACNCANNNNNNNNCNCCNNNNNCNNNNNNNNANNANNNNNNNNNNCNANNNNNNNNCNCNNNNNNNNNNNNCNNNNNNNNCAANNNNNNNNNNNNNCNNNNNNNNNCNNNNNANNNNNNNNCNNNNNNNNNNNNNNCNNNANNNNNNANNNNNNNNNNNNNCNNNNCNNNNNNNNNCANNNNNNNNNNNCCNNNAANNNCNNNNNCNNNNNNNNNNCNNNNNNNCNANNNNNNNNNNNNCNNCNNNNANCNNNNNNNCCNANNNNCNNMNNNNNCCNNNNNNNNANCNNCNNNCCCNNNNNNNNCNNNMNNNNNNCNNNNNNNNNNANNNNCNNNNNCNNNANNCNNNNNNNNNNNNNNCNNNNNNNNNNACNNNNNNNNNANNNNNNNNNANNNNNNNNNCNNNNATNNNNCNANNNCCANNNNNNNNNNNNANCNNNANANNNNNNCNNAANNANANNNNNNNNNACANNNNNNCNNNACANNNNANNNCNNNCNNNNCNNNANNNNNCNNCANCNMNCNNNNNNNNNANNNNNNNNNNNNNANNNNCNACNAANNNNNNNCNNNNANACNNNNNNNNNACNANNCNNNANANNCACNNNNANNNANANCNNACNNCAANNNCNNAANNANNCNNNACNNCCANNCNCACNNANNNCHACAAACNANNCNCAAACANNAANNANCACNNRTCNCAANCNNNNNCCCNANNTANNAYNNNNNNNNCWANNCCAANNCAAACNAATCTNACNCNCNNCATNNAACNNNNCNNTAACNNANNTNTCNCTGCACANATNCNANANCANNCNTCAATANANCTNTANNTAACCNACACNNNN  >*Gammarus lawrencianus*  AAATACNTCGCAGCATCTATTAAGTTGCTGCGGNTTAAAGGCTCGCAGTTTGAATGTCTGTATCGAGCGCAGGTCAGGTGGACGTTGGGGCGTGGATATA  TGGGTGGTTGGTGGGTAGGATTCTAGCCTCACGGCTGTGGCCTCTCTCCTTCTGCNTTTCCACACACCCCCGTCNGTAANAAGCCTCCNCNCTCGNGTAC  ATAANNGGAANCNNTCANACNGAGNGCNGCCNTGCGCATGTGNNCACNCANGTGNGTGCGATGGCGTTAAACTCCATCNNTTTCNCGGTCACCTTGNGTAAATCACAGNGCNCANAGCACNCNNATNGGCCNTGNGCCCNGTGAANANNCTNGACAGACNGACNTGNNTTTGCATGNNATGTNTNAAAACAACATCTNGCGCTGGCTCACAGAANAAATCACTGNNACAANCCNCCCNCTNCATANTGANTANAANGGACAGTCNAGNGNATTGNTATTGNGAGNCGAGAGGTGAAATTCTNTGACNCCCNCANGACATCNNGAAACGAAAACGNCTGCNNANGANATTTTCATTATTCNNGANCGAAAGTTATAGGATCTNANACAATNACATACCNCCACA  >*Hyalella azteca* clade1  AAANAAATCGCTNNTANCATCTATTAAGTTTGCTGCGGNTTAAAAGGCTCGCAGCTTGGAGCCACTGGTGTTTGGTATATGGAGCAGCTGGACGTTGGGT  ACCTCGTGTCCCCGTCCTGTTGTCTGCTCCTGACTCTATGGCACTGAATATGGGAAAGCCTCGCGCGGCTTGGGCGATTTGCTCAGAGGTGTTGGTGTTG  GGCGATTTTAAGCTTTTTGCTTTGCGGCAATCTGCTTTGCGTTTCAAGCCCCCTCTGTGTCCTTGCTCGCGCGGGGTGGCTCCTGTGTCACCTTGAGTAA  ATCATGGTGCTCACAGCAGTCGTGCGGGCCTCGTGCCCTGTGAACAGTCTTTGCGGACTGAATTGGTTTTGCAAGGCATGACGAAACATGACGTCGCTGC  TGGTCGCGGGCGGAGGGCTTTGCNGCCCCACCGGNTNGNCAGCAGTGACGTAATGATCAATGNAGACGGNAGCTGGCATTGNTATTGCNCGGCGNAGAGGTGAAATTCTGCNACCCCCNCAAAACCTCCTAAAACGAAGGCNTCTGCCAANGACGTGTTCACNAATCATNAACGAAAATNAAAGGATCCAANACAATNAAATACNGTCATANACTNTTNTNNAAAAAAAAAACNAAAANANTCAAANAATNAAAAATANNTATNAAAAAAACAANANCAANNAACNAAANNAANTNCTNAAATCAGAATAAAAANAATAAATNAAAAANTAAANAANNACANANAATAANAATAAAAANTAATAAANNAAAAAANNAATCNTNAAAACCNAAAAAANCTAAAANANNANNNNAAATAAAAANANNAAANNACAACNAANAANAANAAAANNANCATAANATAACATCTAAAANTTANTNAAAACACNCAAAAAANNTCAAACCNNANAATATCGATATAAANTATNAACNNTNAAAAAAAATANAAANCAAANAAACNNTATCNNAATAANAAAATANAACNANATCCATCTAAATAGTTAANNNAANAATTAAAAAANNATTAAANATTAAATATAAAACNTNANANAAAAANCTTNAAATANAAAANNTATAAACNAAANAAAANNACATNTCNNANNNNATCTATAAAATNAGAAAAANCCATAANATAAANAAATAAATAAAANCANATAAACCAAAAAACNTNNATATAAATAAATANTNA  >*Hyalella azteca* clade8  NAANCCNTCGTAGCATCTATTAAGTTGCTGCGGTTTAAAAGGCTCGCAGTTGGAGCCACTGGGTGTTTGGTATATGGAGCAGCTGGACGTTGGGTACCTC  GTGTCCCCGTCCTGTTGTCTGCTCCTGACTCTATGGCACTGAATATGGGAAAGCCTCGCGCGGCTTGGGCGATTTGCTCAGAGGTGTTGGTGTTGGACGT  TTTGAGGGTTTATCTCTCTTTTCGTTTCAAGCCCCCTCTGTGTCCTTGCTTGCGCGGGGTGGCTCCTGTGTCACCTTGAGTAAATCATGGTGCTCACAGC  AGTCGTGCGGGCCTCGTGCCCTGTGAACAGTCTTTGCGGACTGAATTGGTTTTGCAAGGCATGACGAAACATGACGTCGCTGCTGGTCGCGGGCGGAGGAAGATTTGTGTGCTCCGGCGCGCATTCTCTCTTACCGGTGTGTTTATGCCAGCAGCGACGTAATGATCAATGGAGACGGTAGCTGGCATTGGTATTGCGCGGCGAGAGGTGAAATTCTGCNACCCCCGCAAGACCTCCTAAAGCGAANGCGTCTGCCAAGGACGTGTTCACCAATCATGAACGAAAGTCANAGGATCGAAGACGATCANATACCGCCAANNNCNNNATNTNNANNANNANNANNNANANANAAAANANANNANAAANAAAGTANAAANNNNANNAANAANTNANAATNAANANNNNNCANAAAAAAAANANAANAAACAGAANANNANANACACANANNAAAANAANNNAAACAAANNATANTNANAANAAAAATAAAANAAANAANAAANAANNAANTAAAAAAAAAANNNAAAAAAATAAAANAAANANTNANTNTNNATANATNANNAANANNTNAAAAAAAAAAACNGANTAAAANANAAAAANANNAANCANCAAAANNAAANANTAANAANANNANAANNANAANNATAAGAAANNNATNAAACNNAANATTANANAAAAAAANAANCAATANNNNANANANNNNNTNTNANAANANNTNNNANAANNACNAAAAATATAANAANANAAAATAACANTAANANCANAANAATNAATAANNCATAANAAAANACTNTAAAANAAANGATANANTAAANNNNNNTTAAANNNAANANNACAAAATANAANANAAAAACNAAAAAAANNANANTAANNNANTATATTNAANANNANANNAAA |
| >*Limacina helicina*  AGAAATCGCTCATAGCGTATATAANGTTTGTTGCAGTTTAAAAGCTCGTAGTTGGATCTCAGGTGCAGGCGGGCGGTCCGGCTCGCGCCGGTCACTGCTC  GTATCTCCTGCCCTACCGTTTGCCGGCTCTCTCCCGTGGGTGCTCTTCACTGAGCGTCCCGGGTGGCCGGTGCGTTTACTTTGAAAAAATTAGAGTGTTC  AAAGCAGGCCTCGGCAGCCTGAATAATGGTGCATGGAATAATGGAATAGGACCTCGGTTCTATTTTGTTGGTTTTCGGAACGGAGGTAATGATTAACAGG  GACAAACGGGGGCATTCGTATTGCGGCGTTAGAGGTGAAATTCTTGGATCGCCGCAAGACGAGCTACTGCGAAAGCATTTGTCAAGAATGTTTTCATTAG  TCAAGAACGAAAGTCAGAGGCGCGAAGACGATCAGATACCGCCANNTNNNTNTANTTCNTTNNNTNTTTNNNNCNNAANNAANTANNNAACTATNACANNANATNNNANNAANTNNTNNTNNNTNCNTATNCNAANATTNNNATNAACTAAANNATCACAACANANAANNTAAAAAATNTATTANNNTNNNTANATAAANTTANTCNNATCACTCAANCNAATANTNNNCTNATNNAACNANNNTTTNTAAANAANNNTANNTNAANACANATANACATTNNNNTANNNATNNNACATCCNTTCANNNTTTCANNANTNATANATNANNATATTAATNNANNNNANNNATATNAAAATCNCAAAACANAANTAAAATAANNACTACNACCTATNATATCATNACNCANTAANTNNAACTTANNATNANAANACNANATNNNNTNTNNNAACTACTNCAACNATNNCNCCANCANANNANATTATTATAANAANCNNNCNATAAANANNNAAAANTNNANNTTAACTTTTCTATACCNTTNNTAAATATTNANNNNNNCTTATACNATAANANANATNNANANNNTNTAATCTANTCANNNTCATNNTNNATNNTAAANAAATNATNACNNNANCCNTANANNATTNTCNTNNCTANAAAAAAAATANAAAANCTCNCCANANNTCTTNNTTTNCTNNANNCTNCNTTTTNNNCTNNTTACAATNANTNNNNTTATNNNTTAANCANTAANANNAAACTNNNNTGTTAANTCCNANTTTANACTNNTACNCNNNCTTNNCTANTNCANTAANNNACTTTTNCNTTCNTNAATTCCACTCATANNCTNNTATATAACNNATTAATNATNNTTATATCNNT  >*Limnoperna fortunei*  NAAAATCGCTCATAGCGTAATTAAGTTTGCTGCAGTTTAAAAAGCTCGTAGTTGGATCTCGGGTCCAGGCTTGTGGTCCGCCGCTGGGCGGTTACTGCTC  GTCCTGACCTACCTCCCGGTTTGCCCTTGGTGCTCTTGACTGAGTGTCTCGGGTGGCCGGAACGTTTACTTTGAAAAAATTAGAGTGTTCAAGGCAGGCA  ATTCGCCTGTATAATGTTGCATGGAATAATGGAATAGGACCTCGGTTCTATTTTGCTGGTTTTCGGAGCTTGAGGTAATGATTGAGAGGGACTGACGGGG  GCATTCGTATCACGGTGTTAGAGGTGAAATTCTTGGATCGCCGTAAGACGCACTACTGCGAAAGCATTTGCCAAGCATGTTTTCATTAATCAAGAACGAA  AGTCAGAGGTTCGAAGACGATCAGATACCGNCA  >*Majidae*  GNAAATCGCTCAAANCGNANATTAAAGGTTGTTGCGGTTTAAAAAGCTCGTAGTTTGGATTTCAGNTTCTGGACTGACGGTTCACCGCCCGGATGCACAC  TGTCACGCTCCGAACAGCCACAACAGCCCGCTGGCTCGCACGGGGTGCTCTTCATCGAGTGTCCCGCGTGGCCGGCAGAGTTTACTTTGAAAAAATTAGA  GTGCTCAAAGCAGGCTACACTGACGGCCTGAATGCCTATGCATGGAATAATGGAATAGGACCTCGGTTCTATTTTGTCGGTTTTCTGAACCCGAGGTAAT  GACTAATAGGAACAGGCGGGGGCATTCGTATTGCGACGCTAGAGGTGAAATTCTTGGACCGTCGCAAGACGAACTACTGCGAAAGCATTTGCCAAGGATGTTTTCATTAATCAAGAACGAAAGTTAGAGGTTCGAAGACGATCAGATACCGCCANTNNTNTGNNTCAAANNACANCNTAAANTANTNNANTNNATANNANACNATNATNNNNACTNACNACANNAAATNTACCACACNTGNACANCNANNAAAACNAANANNNNNATACGNANTCAAANACTTACTNTNTNAANTNTNATAANTNNNNANAACNAANNCTNAATNCAANTNNATAAATACATNNNNNNANTAATTNNAANNANAGNAANNTANANNNTATTNNNATAANATCNNANCTANNNCNNANGTCNAANTNNTCTNCTTTNAATNACANNNNTAAACACNNNCANTNATTTTACNACCCNNGNCNANNACNATATCTANAATTATCAAANNANNNTAANCAANNTTTTANCNAAAANTNATTANTTNCATANANAANTTNNNATTAGNANCATNCANANNCANTNNNNAACCTNCAANTAANTNAAANTNCNTTCNNNCACTTNNAANGNGATAACCATAATATACNCNNCNNANAANTNACTNAANNNCANNTNCANCAAGNATACNTTCGATNNCANCNCTTTANNTCAAANANNNNCANNANANNTACNAATNCNATNNTANACNNACGCTTNCANAATANANNTNNAAACNCANNAAACTAANTNNNCANANAAACTANNNTNNNTATNNTCANCCCANTAATNACNNAAATNCCNNNGCANACAACANATCNCNTNACACNCCCANNATANANNNAATTCCANNACAANNNTNNNNNCACATCTNTNAAACANCNAANNANNCTATACATANTCCNACAANTNNNNNTANTNCGTTNCNGNAACATATANACTCTNNATANNNNCNACGCNTATAAANNCATNNNCTNCCTTT  >*Mesocyclops edax*  NAAAATCGCTCATAGCGTAATTAAAGTTGTTGCGGTTAAAANGCTCGTAGTTGGATCTCGGCGGGCAGGGGGCGGTCCGTTATTTGACGCGACTGCCCCG  TTTGACCGTGTTTTTTTGGTGGAACTCGCAGGGTGCTCTTCGCCGAGTGTCCTGTCGGGGCCATCGGGTTTACTTTGAAAAAATTAGAGTGCTCCAAGCA  GGCATGATACGCCTGAATATCCGTGCATGGAATAATGGAATAGGACGTCGTTCCTATTTTGTTGGTTTTAGGGAATCGACGTAATGATTAATAGGGACAG  CCGGGGGCATTAGTATTCAGACGACAGAGGTGAAATTCTTGGACCGTCTGGCGACTCACTGCTGCGAAAGCGTTTGCCAAGTATGTTTTCATTAATCAAG  AACGAAAGTTAGAGGTTCGAAGACGATCAGATACCGCCANNNNNNNNNNNNNNNNNNNNNNNNNNNNNNNNNNNNNNNNNNNNNNNNNNNNNNNNNNNNNNNNNNNNNNNNNNNNNNNNNNNNNNNNNNNNNNNNNNNNNNNNNNNNNNNNNNNNNNNNNNNNNANNNNNNNNNNNNNNNNNANNNNNNNNNNNNNNNNNNNNNNNNNNNNNNNNNNNNANNNNNNNNNNNNNNNNNNNNNNNNNNNNNNNANNNNNNNNNNNNNNNNNNNANNNNANNNNNNNNNNNNNNNNNNNNNNNNNNNNNANNNNNNNNNNNNNNNNNNNNNNNNNNNNNNNNNNNNNNNNNNN  NNNNNNNNNNNNNNNNNNNNNNNANNNNNNNNNNNNNNNNNNNNNNNNNNNNNNNNNNNNNNNNNNNNNANNNNNNNNNNNNNANNNNNNNNNNNNNNNNNNNNNNNNNNNNNNNNNNNNNNNNNNNNNNNNNNNNNNANNNNNNNNNNNNNNNNNNNANNNNNNNNANNNNNNNNNNNNNNANNNNNNNNNNNNNNNNNNNNNNNNNNNNNNNNNNNNNNNNNNNNNNNNNNNNNNNNNNNNANANNNNNANNNNNNNNNANNNNNNNNNNNNANNNAANNNNNANNNNNNNNNNCNAANNNNNNNNNNNNNNNANNNNNNNNNNNNNNNNNNNNNNCACNCTATNNNC  CNCCTANACNCAATACAATANATCCNNACCAGANTACNNTCAATACNACNCTNTGATANATCANTAATAGTACAACNTCNAACAAGNANNTNNCNANCANCTCANAACANANNTNACTACACNNNNNTNNTACATCAATAGNTNNNCNNANNNNNNATATTAANAATNTTNNTAGANTTGGCNCTNNCCANACNTANCCCAA  >*Microsetella norvegica*  ANNAANCGCTCATAGCGTANTTAANGTTTGTTGTGGTTAAAAGCTCGTAGTTGGATCTTGGAAGGTCGGGGGCGGTGAGGTGAATAGCCGCACTGCCCCATTGGCTCTTCTGACATTGGGCGGCAACCGGCTGGTGCTCTTAACCGAGTGCTAGACATCGGCGCCGCCAGGTTTACTTTGAAAAAATTAGAGTGCTTCAA  GCAGGCTTCAAACGCCCGAATACTCGTGCATGGAATAATGGAATAGGACGTCGTCACTATTTTGTTGGTTTTCGGAGATCGACGTAATGATTAACAGGGA  CAGTCGGGGGCATTGGTATTGAGGAGCTAGAGGTGAAATTCTTGGACCTCCTCAAGACCAACCACTGCGAAAGCATTTGCCAAGAATGTTTTCATTAATC  AAGAACGAAAGTTAGAGGTTCGAAGACGATCAGATACCGCCANANGNNNNNNNNNNNCNNGANANTNNNANTATNACNNNNCAACCAANNANNAAAAATANNNNNNNNANANGCNNTNANNAANAAATCAANNAANNNANTNANANNANAAAATTNNANTGTNTNNAANAAANCNAAANATNANANNNTNAAANNANNNNAANANAAANGNTNAANNNNNNNANNNNNTNAACTTAAACTNAAANTTACNANAANANCNNNNNTACNNNNTNNATTNNTNNNCCAAATANNATNTTANNANTAAAACACTGANTNNNNAANTAAANNNNNANATNNAANCNTNTCTNANANAANACNNAAATNANNCNNAANCCNTGANNCTNNNNNGACNAANANNANNTNTTAANNANNAACANNANNNCAATNTANANANCNCNANTNNNNNNANNNNAAAANTNANNCNTTTNNATNCGAACAANNAATGANATNTNNNNNATACANTTNCCNTAANCANNNCTNGCATNAACANNNNANANNANCATNNTANTNANNAAATNAANNNTNAANTACNNANNANAAATNAAANNNTNANNANTNNNTNANACNNACCNNNNATANATANTNTCNANAAATNTNANANNCATNACAANTCNCAANANATNTNAAACCNNNNTTTNAANACANNATATNANTATAGCTANANACNNANANTNAANTTNANTNNTNNTNCCNAANAACNNANNCNAAAANNCNNNAANNANNNCTAANANCTNNAANATNTAANNNNACTACTNNCC  >*Nerita spp.*  AAAAAATCGCTCATAGCGTATATTAAAGTTTGTTTGCAGTTTAAAAAGCTCGTAGTTTGGATCTCGGGTTTGGGCGGGCGGTCCGCCTCGCGGCGGTTAC  TGCACGACCCGACCTACCTCCCGGTTTTCCCTTGGTGCTCTTGACTGAGTGCTTCGGGTGGCCGGAACGTTTACTTTGAAAAAATTAGAGTGCTCAAAGC  AGGCGCTTCGCCTGAATAATGGTGCATGGAATAATGGAATAGGACCTCGGTTCTATTTTGTTGGTTTTCGGAACTCGAGGTAATGATTAAGAGGGACAGA  CGGGGGCATTCGTATTACGGTGTTAGAGGTGAAATTCTTGGATCGCCGTAAGACGAACTACTGCGAAAGCATTTGCCAAGCATGTTNTCATTAATCAAGA  ACGAAAGTCAGAGGTTCGAAGACGATCAGATACCGCCAANTNNNTTTNNANANTNTCANCNTNTTANNCATNTNATTCTNTAATCAANCCNNCCNAACNNNACTNANNATATATACNTTNTNANACCANCCAANCNCTANAAAAANATCNATAATNNANCNNACATCNTCNTTCNTACNTCANNNATNNNCNANNNTNTCACAAANCAATNCNNATNNATANNACNCNATNANATNTNNNATTAANAATNCAANTCNAANNNACNANCNNNAANTAAAAANTACTTNCACATNAACTATNNNATTNCTNANNCNNCATANNNAATAANNCNCATANANCNTNTCAANAATACACTAANNANNCNTNTTCTNTCNATANTACTNAAAANCNACANNACNNTAATATAATAACNNTAANCATTNCNATCATNNNCCTNCAACATAATNNANNNCTTCATNATNAAAAATTACNNANNAATNAAACAANANACNAATNNTACTTCTNNNCNATAANNNNAATACATNNAANAANNANNATCNACAATNNNACTNNTAANNAAANANAACAATAATNNANACAACNAACATAAACNCANNAAANNNAANNCTNANCNACANNNTAATAACANTNNNANANTATCTANCNNANTCTNNNTTACAANATATNATNNAAANTAATCANNANANATNTTTAACNACCNTTATATNNNATNNATNTCNTNANGNNACNANANCTANCCNNTANANTANNNCANANNNNNNCNANTTNANAATTATCNANATAAACAANNNNNNNAAGNNANNNTACTNAATTANANTCNCANNATNANNNNCANT  >*Oikopleura labradoriensis*  CGCTCAAAGCGTANCTAAGTTTGGTTGCGGTTAAAAGCTCGTAGTTGGATCTTGGAGCGTGTGCTTGCGGTCACATTTCGGTGTGTACTGCTTGACGTGT  TCTTCTATTTGGTTGCGCGTGTGTGTGCTCTTACCTGANTGCCGCATTCGACCGAAACGTTTACCTTGAAAAAATTANAGTGTTCAAAGCAGACCTTTTT  GTGTCTGAATATTCGTGCATGGAATAATGAAATATGAATTCGGTTCTATTTTGTTGGTTTTCGGAACTCGAGTTAATGATTAAGAGGGACAGACGGGGGC  ATTCGTACTCTGCCGTTAGAGGTGAAATTCTTGGATCGGCGGAAGACGCGCAACTGCGAAAGCATTTGCCAAGAATGTTTTCTTTAATCAAGAACGATAG  TCGGAGGTTCNAAGACGATCAGATACCGCCACACNCNNCNNCCAAATNANNNAGNCACNTNNANANNAAACACNNCNNNAANCNNNATNCNCANAANAAACNCNANAGAANAGANAANATNNCTACNAANANACAANCACANANAAANAANAATAANNCGATCTAAANAGNCGNCANANNANAAAAAAANNNNNACNANAAANANNAANAAANCNAATNNGNANNGAAAATNNCAAANACANCNAANCCATGGNNAANCANCCNCNAATNANNCATATACNANNATNNNTNACNNCANANNNAACAACACCNNANACCGCAATNNNNCCCAANANCANCATAAGANATNCCNTANACAAANAAANNCNNCAACNCNCNTNCCNACANNTNCNAATNNCCAANNAANAAGTNNNCAANNNAANNACNANNNNNNATCNGNGNAAAAATAANNNCNTCNACAANAACNNANAGACCNCTANAANNNNANGCANNNAAAANNANANAAANNAAAANGCNTCNANNAAGNNAANANCNCNANGNCATNANNACTACNNACANACANAATNAACAANNNNNAAATANAAAGNAAACCANNCACCAAAAAAAACCACTCATCANCAGANACGTCNNNNAACANCNANAANCAACAGCATNATCATACCAGNAANACATACCCNAANAAGGNCAGNGANNANCAANCNAAANNTCANNAACNANGACACAGCCGANGANACCANAACATNNGAANTANNCTCNACGNNCNNCTACNNTNAGNAGNNNTGAACGCATATCNTGNNACAAAACAATAGCCNCNNTTCCACGANCTANANAGCATANAACAAACANTAGTACTCATNNANACNNCNGATCA  >*Pteropod*  AAANAAAANCGCCNAAAGGCGTATATAAAAGTTTGGTTGCAGTTTAAAAAGTCTCGATAACTTTGAGGATCTCAGNGTTGGCAGGCCGGGCGGNTCCGGCTCGCGCCGGATCACTGCTCGTATCTCCTGCCCTANCGCTTTGCCGGCTCTCTCCCGTGGGTGCTCTTCACTGAGACGTCCCGGGTGGCCGGTGCGTTTAC  TTTGAAAAAATTAGAGTGTTCAAAGCAGGCCTCGGCAGCCTGAATAATGGTGCATGGAATAATGGAATAGGACCTCGGTTCTATTTTGTTGGTTTTCGGA  ACGGAGGTAATGATTAACAGGGACAAACGGGGGCATTCGTATTGCGGCGTTANAGGTGAAATTCTTGGATCGCCGCAAGACGAGCTACTGCGAAAGCATTTGTCAAGAATGTTTTCATTAGTCAAGAACGAAAGTCAGAGGCGCGAAGACGATCAGATACCGCCANNNNNCACTNNTGTTNAATAAAANAAAAACTTNGGANANNANTNTCNANCTNAAANNNANNATACANNNNTTATNANCNCANTAACAGAAACNAANNANNAGCTTNCANCANATNTCNATTNNCCANAGATNCNCCANTTACATTNAAAANCANAAAAATNAANAANANANACNAAACTANACAATCAANNAAATNCNCACNAAANNACTTNANANNTCNNNNTTATAANNNNAANTNNCACCATGTANCACNNANNNANATNAAAANNNAAGAACTAANNNCANTCNAACNAANCNNTANANANAANANTAAANATAANAANNNNATNAAAGANTNAAACNTAACNNCANNNAAAACNACNACTANACANANCAAAGNAANANTNTAANTATNATNAATANAAACNTTNNNTNANCCACTATCTATTANNNTAAANANATAAAANTTNNATNNNNAANNAANNANTAATTTNNNNNCANNANAAANATNTAANAAACANNNNNANNNATCNNNATANNNANTTANNCNANANANNTCNTTNTCTACTNTACATCCCNANANCNTNTNNNANACTATCNANCTACANNNNNAATAAANCCTNCTAANNANCATTACATNNTAAANANTAATNNNCAAATCNCCGCNANNNNAACGTATAAANNNTNNCACAAANTNNANNNNNATANTTANANCATTNACGNANNTACANNAATAANNCAANNAANNACTAANCNAANCATNNCANAAANNNNAATNNATAAATCTNNTCNNACATCNANTCNTCTACTNAAANCANNANCNCTANANTNTNNATAAANNCGATNNCANAAATCAATCANNAAACNTNNNNNTACTACAATTAACNNCTTTATGNNACAANCACACNNA |

Table S6. The number of OTUs generated and species detected when clustering at 3% (with singletons either excluded or included) compared to the number of expected species, for each genus or family included in the Untagged Individuals Community. The expected number of species was determined by BLASTing raw reads to verify the species that were successfully amplified. The number of OTUs generated does not always correspond to the number of species detected, since multiple OTUs may be generated for a single species. The number of species detected may not match the expected number of species if closely related species are collapsed into shared OTUs (e.g. *Limnocalanus*). Species detected only when singletons are included within the dataset are highlighted in grey.

|  | Expected no. of species | Singletons excluded | | Singletons included | |
| --- | --- | --- | --- | --- | --- |
| Genus/family |  | No. OTUs generated | No. species detected | No. OTUs generated | No. species detected |
| *Acartia* | 1 | 1 | 1 | 1 | 1 |
| *Artemia* | 2 | 1 | 1 | 1 | 1 |
| *Balanus* | 2 | 1 | 1 | 2 | 2 |
| *Bosmina* | 1 | 1 | 1 | 1 | 1 |
| *Bythotrephes* | 1 | 1 | 1 | 1 | 1 |
| *Calanus* | 1 | 1 | 1 | 2 | 1 |
| *Caridea* | 1 | 1 | 1 | 1 | 1 |
| *Centropages* | 1 | 1 | 1 | 1 | 1 |
| *Chthamalus* | 1 | 0 | 0 | 1 | 1 |
| *Ciona* | 1 | 1 | 1 | 2 | 1 |
| *Clytemnestra* | 1 | 1 | 1 | 1 | 1 |
| *Corbicula* | 1 | 2 | 1 | 4 | 1 |
| *Corycaeus* | 1 | 1 | 1 | 1 | 1 |
| *Crangonidae* | 1 | 1 | 1 | 2 | 1 |
| *Crangonyx* | 1 | 1 | 1 | 1 | 1 |
| *Daphnia* | 3 | 3 | 2 | 4 | 2 |
| *Diaphanosoma* | 1 | 2 | 1 | 2 | 1 |
| *Eucyclops* | 1 | 1 | 1 | 1 | 1 |
| *Eurytemora* | 1 | 2 | 1 | 2 | 1 |
| *Gammarus* | 3 | 3 | 2 | 6 | 2 |
| *Grapsidae* | 1 | 1 | 1 | 2 | 1 |
| *Holopedium* | 1 | 1 | 1 | 1 | 1 |
| *Hyalella* | 2 | 4 | 2 | 6 | 2 |
| *Hyperia* | 1 | 1 | 1 | 1 | 1 |
| *Hyperoche* | 1 | 1 | 1 | 1 | 1 |
| *Leptodiaptomus* | 1 | 1 | 1 | 1 | 1 |
| *Leptodora* | 1 | 2 | 1 | 2 | 1 |
| *Limacina* | 1 | 1 | 1 | 1 | 1 |
| *Limnocalanus* | 1 | 0 | 0 | 0 | 0 |
| *Limnoperna* | 1 | 1 | 1 | 1 | 1 |
| *Macrocyclops* | 1 | 2 | 1 | 2 | 1 |
| *Mytilus* | 1 | 1 | 1 | 1 | 1 |
| *Nassarius* | 1 | 1 | 1 | 1 | 1 |
| *Neotrypaea* | 1 | 1 | 1 | 2 | 1 |
| *Nerita* | 1 | 1 | 1 | 1 | 1 |
| *Oikopleura* | 1 | 1 | 1 | 1 | 1 |
| *Polyphemus* | 1 | 1 | 1 | 2 | 1 |
| *Pseudocalanus* | 1 | 2 | 1 | 2 | 1 |
| *Themisto* | 1 | 1 | 1 | 1 | 1 |
| *Tisbe* | 1 | 1 | 1 | 1 | 1 |
| *Zaus* | 1 | 1 | 1 | 1 | 1 |
| Total | 49 | 51 | 41 | 68 | 44 |

Table S7. Species detected, listed by Genus or Family, when clustering data generated by the Untagged Populations Community at 3% when singletons are either excluded or included. The expected number of species was determined by BLASTing raw reads to verify the species that were successfully amplified.

|  |  | Singletons excluded | | Singletons included | |
| --- | --- | --- | --- | --- | --- |
| Genus/family | Expected no. of species | No. OTUs generated | No. species detected | No. OTUs generated | No. species detected |
| *Artemia* | 1 | 2 | 1 | 2 | 1 |
| *Balanus* | 1 | 1 | 1 | 1 | 1 |
| *Carcinus* | 1 | 1 | 1 | 1 | 1 |
| *Chthamalus* | 1 | 1 | 1 | 3 | 1 |
| *Corbicula* | 1 | 2 | 1 | 2 | 1 |
| *Daphnia* | 1 | 1 | 1 | 2 | 1 |
| *Eurytemora* | 1 | 1 | 1 | 2 | 1 |
| *Gammarus* | 1 | 0 | 0 | 0 | 0 |
| *Hyallela* | 1 | 3 | 1 | 5 | 1 |
| *Leptodiaptomus* | 1 | 1 | 1 | 1 | 1 |
| *Leptodora* | 1 | 1 | 1 | 2 | 1 |
| *Nerita* | 1 | 1 | 1 | 1 | 1 |
| *Palaemonetes* | 1 | 3 | 1 | 5 | 1 |
|  | 13 | 18 | 12 | 27 | 12 |

Figure S1. Sequence quality (PHRED quality score) per base position for the Untagged Individuals Community dataset, as reported by FastQC (Andrews, 2010). The blue line represents the average quality, the yellow box represents the inter-quartile range and the upper and lower whiskers represent the 10% and 90% points. The three other datasets analysed show similar distributions.


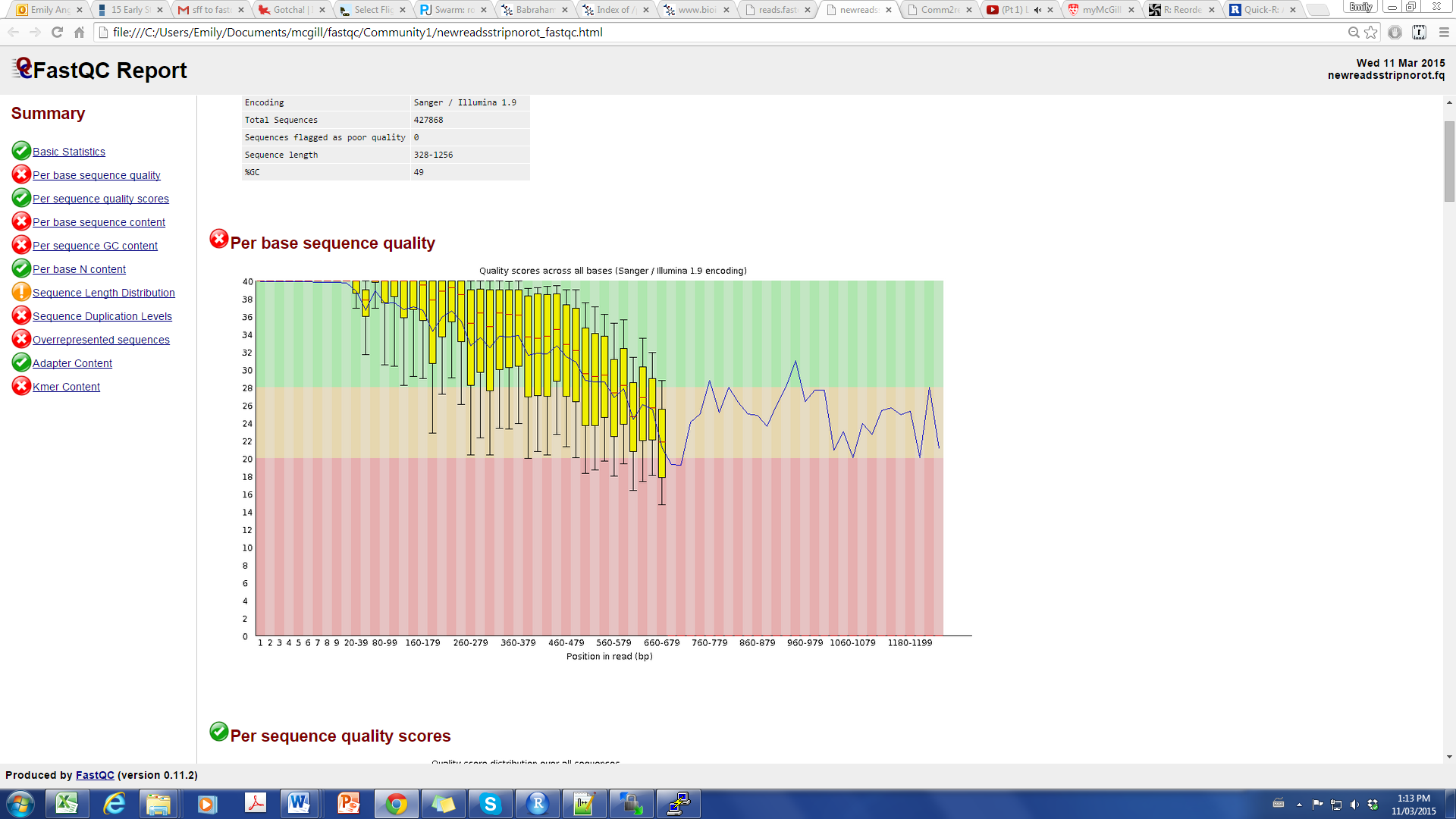


**Reference**

Andrews S (2010) FastQC: a quality control tool for high throughput sequence data. Available online at: <http://www.bioinformatics.babraham.ac.uk/projects/fastqc>
